# PUL21a-Cyclin A2 Interaction is Required to Protect Human Cytomegalovirus-Infected Cells from the Deleterious Consequences of Mitotic Entry

**DOI:** 10.1371/journal.ppat.1004514

**Published:** 2014-11-13

**Authors:** Martin Eifler, Ralf Uecker, Henry Weisbach, Boris Bogdanow, Ellen Richter, Lydia König, Barbara Vetter, Tihana Lenac-Rovis, Stipan Jonjic, Heidemarie Neitzel, Christian Hagemeier, Lüder Wiebusch

**Affiliations:** 1 Labor für Pädiatrische Molekularbiologie, Charité Universitätsmedizin Berlin, Berlin, Germany; 2 Center for Proteomics, Faculty of Medicine, University of Rijeka, Rijeka, Croatia; 3 Institut für Medizinische Genetik und Humangenetik, Charité Universitätsmedizin Berlin, Berlin, Germany; University of Wisconsin-Madison, United States of America

## Abstract

Entry into mitosis is accompanied by dramatic changes in cellular architecture, metabolism and gene expression. Many viruses have evolved cell cycle arrest strategies to prevent mitotic entry, presumably to ensure sustained, uninterrupted viral replication. Here we show for human cytomegalovirus (HCMV) what happens if the viral cell cycle arrest mechanism is disabled and cells engaged in viral replication enter into unscheduled mitosis. We made use of an HCMV mutant that, due to a defective Cyclin A2 binding motif in its UL21a gene product (pUL21a), has lost its ability to down-regulate Cyclin A2 and, therefore, to arrest cells at the G1/S transition. Cyclin A2 up-regulation in infected cells not only triggered the onset of cellular DNA synthesis, but also promoted the accumulation and nuclear translocation of Cyclin B1-CDK1, premature chromatin condensation and mitotic entry. The infected cells were able to enter metaphase as shown by nuclear lamina disassembly and, often irregular, metaphase spindle formation. However, anaphase onset was blocked by the still intact anaphase promoting complex/cyclosome (APC/C) inhibitory function of pUL21a. Remarkably, the essential viral IE2, but not the related chromosome-associated IE1 protein, disappeared upon mitotic entry, suggesting an inherent instability of IE2 under mitotic conditions. Viral DNA synthesis was impaired in mitosis, as demonstrated by the abnormal morphology and strongly reduced BrdU incorporation rates of viral replication compartments. The prolonged metaphase arrest in infected cells coincided with precocious sister chromatid separation and progressive fragmentation of the chromosomal material. We conclude that the Cyclin A2-binding function of pUL21a contributes to the maintenance of a cell cycle state conducive for the completion of the HCMV replication cycle. Unscheduled mitotic entry during the course of the HCMV replication has fatal consequences, leading to abortive infection and cell death.

## Author Summary

Cyclin A2 is a key regulator of the cell division cycle. Interactors of Cyclin A2 typically contain short sequence elements (RXL/Cy motifs) that bind with high affinity to a hydrophobic patch in the Cyclin A2 protein. Two types of RXL/Cy-containing factors are known: i) cyclin-dependent kinase (CDK) substrates, which are processed by the CDK subunit that complexes to Cyclin A2, and ii) CDK inhibitors, which stably associate to Cyclin A2-CDK due to the lack of CDK phosphorylation sites. Human cytomegalovirus (HCMV) has evolved a novel type of RXL/Cy-containing protein. Its UL21a gene product, a small and highly unstable protein, binds to Cyclin A2 via an RXL/Cy motif in its N-terminus, leading to efficient degradation of Cyclin A2 by the proteasome. Here, we show that this mechanism is not only essential for viral inhibition of cellular DNA synthesis, but also to prevent entry of infected cells into mitosis. Unscheduled mitotic entry is followed by aberrant spindle formation, metaphase arrest, precocious separation of sister chromatids, chromosomal fragmentation and cell death. Viral DNA replication and expression of the essential viral IE2 protein are abrogated in mitosis. Thus, pUL21a-Cyclin A2 interaction protects HCMV from a collapse of viral and cellular functions in mitosis.

## Introduction

HCMV (also referred to as human herpesvirus-5, HHV5) is widely distributed in the human population. Acute HCMV infection can cause severe complications in immunocompromised individuals, like neonates, transplant recipients and AIDS patients. Persistent HCMV infection has been implicated as a contributing factor in the complex etiology of chronic disorders like inflammatory bowel disease, atherosclerosis and cancer [1], [2]. Treatment of HCMV is limited by severe side effects of available virostatics and by the emergence of resistant strains [3].

At the cellular level, HCMV can establish either a latent or lytic, productive infection, depending on the cell type and differentiation status. Lytic HCMV infection is accompanied by dramatic changes in host cell physiology, which are induced by the virus to promote its replication and dissemination. To this end HCMV has evolved an arsenal of regulatory factors that interact with central control mechanisms of the host cell. Besides metabolic pathways [4], cell death programs [5], intrinsic and innate immune responses [6], one of the main targets of HCMV is the cell division cycle [7], whose proper function is essential for the maintenance of genomic stability and cell growth control.

Progression through the somatic cell cycle relies on the periodic activation of cyclin-dependent kinases (CDKs) [8]. Fundamental to this periodicity is the temporal and spatial regulation of cyclin proteins, which are required for both CDK activation and substrate recognition. In brief, mitogen-dependent induction of Cyclin D1–CDK4/6 activity in the early stages of the cell cycle (G1-phase) leads to phosphorylation of the retinoblastoma protein (pRb) family of transcription factors and hence to a de-repression of growth-promoting, pRb–E2F-controlled genes, including those encoding E, A and B-type cyclins. Increasing levels of Cyclin E1–CDK2 activity in late G1 trigger S phase entry by further up-regulation of pRb–E2F-dependent gene expression and by promoting the process of replication licensing [9], [10]. In S phase, Cyclin E1 is marked for proteolysis by SCF-dependent ubiquitination [11], [12], whilst Cyclin A2 and B1 proteins become stabilized by inactivation of the APC/C ubiquitin ligase [13]. Cyclin A2–CDK1/2 activity catalyzes the initiation and regular progression of cellular DNA synthesis as well as entry into mitosis [14]–[18]. The importance of Cyclin A2 for the G2/M transition is in large part due to orchestrating regulatory processes controlling the major mitosis-promoting factor, Cyclin B1–CDK1 [19]–[24]. This complex is held inactive until G2/M transition by inhibitory phosphorylation of CDK1 and cytoplasmic localization of Cyclin B1 [23], [25]. Once activated, it drives mitosis up and through metaphase. Further transition to anaphase can only occur after relief of the spindle checkpoint-mediated restriction of the APC/C. As a consequence, Cyclin B1 is rapidly destructed to allow mitotic exit [26]. Evidence from conditional knockout mice suggested that CDK1 has a ubiquitous essential role for cell division [27]. Cyclin A2 is essential in embryonic and hematopoietic stem cells but not in mouse embryonic fibroblasts where its loss can be compensated by E-type cyclins [28]. On the other hand, combined conditional knockout of Cyclin A2 and CDK2 is sufficient to impair fibroblast proliferation [29]. This correlates with the non-overlapping role of Cyclin A2 in many cultured somatic cell lines [14]–[18], [19]–[24], [30].

HCMV disturbs the regular order of events in the cell cycle control machinery. It represses Cyclin D1 [31] and instead employs viral gene products to achieve pRb hyperphosphorylation [32], [33] and E2F target gene activation [34]. It induces Cyclin E1-CDK2 activity [35]–[38] but at the same time inhibits replication licensing [39]–[41]. It inhibits the APC/C ubiquitin ligase leading to the accumulation of numerous APC/C substrate proteins including Cyclin B1 [42]–[44]. However, HCMV blocks expression of the APC/C substrate Cyclin A2 [44]–[46]. Moreover, Cyclin B1-CDK1, although expressed and activated, is retained to the cytoplasm unable to trigger mitotic entry [47]. The net effect of these HCMV-induced alterations is a unique cell cycle state, characterized by a block of cellular DNA synthesis and cell division in the presence of a fully induced nucleotide metabolism as well as up-regulated replication and repair factors. Such conditions are considered to be most favorable for efficient replication of the HCMV genome [7], [48], [49].

Before HCMV can take control over the cell cycle, it is itself subjected to cell cycle-dependent regulation [50]. In the pre-immediate early phase of infection, i.e. the time between virus entry and *de novo* expression of viral gene products, HCMV is blocked by Cyclin A2-CDK2 activity [51], [52]. This block relies on a Cyclin A2 binding site in the HCMV inner tegument protein pp150 [53], matching the RXL/Cy consensus motif found in cellular Cyclin A2-CDK substrates and inhibitors [54]. Apparently, HCMV has acquired this motif to make the incoming virus particle sensitive to cellular Cyclin A2, thereby restricting the start of lytic gene expression to cells with low or absent Cyclin A2-CDK activity like quiescent, differentiated and G1 cells [53]. Besides this general effect on lytic gene expression, Cyclin A2-CDK interferes more selectively with expression of the essential IE2-86 kDa protein [51]. This might be one of the reasons why HCMV suppresses Cyclin A2 once the lytic gene expression has started [44]–[46].

In this study we aimed to deepen our understanding of HCMV-Cyclin A2 interaction by investigating the cause and consequences of Cyclin A2 repression during lytic infection. We found that HCMV encodes a second RXL/Cy motif that targets the highly unstable pUL21a protein [55] to Cyclin A2, resulting in its proteasomal destruction. This mechanism turned out to be of crucial importance for the HCMV-induced cell cycle arrest, preventing the virus from mitotic entry and resultant abortive infection.

## Results

**Figure 1:**
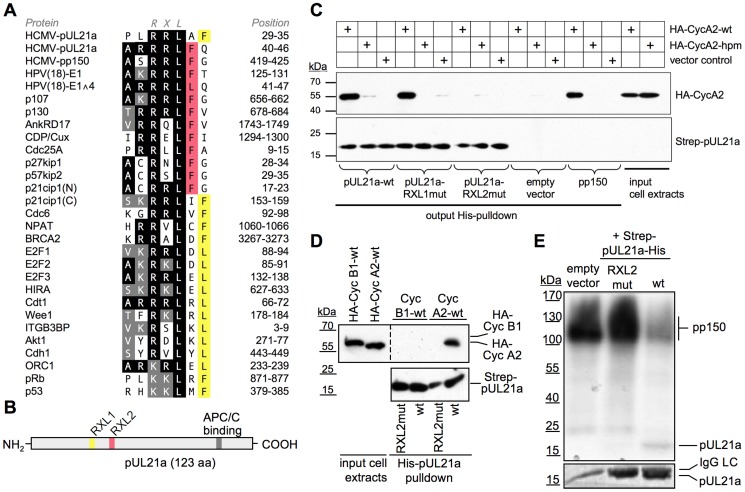
HCMV-pUL21a interacts with Cyclin A2. (A) Alignment of two putative Cyclin A2-binding sites in pUL21a with validated RXL/Cy motifs of human Cyclin A2-CDK substrates and inhibitors. Identical residues are highlighted in black, conserved residues in grey, phenylalanine/leucin residues at positions +1 or +2 relative to the RXL motif in red or yellow. (B) Schematic of pUL21a showing the relative location of RXL sequence motifs, the APC/C-binding site and a minimal consensus CDK phosphorylation site (SP). (C, D) Recombinant Strep-His-tagged versions of pp150, pUL21a wild-type (wt), pUL21a-RRLAF^ARAAF^ (RXL1mut) and pUL21a-RRLFQ^ARAFQ^ (RXL2mut) mutants were purified and immobilized to Ni-NTA agarose beads. The beads were incubated with HEK293 cell lysates containing ectopically expressed HA-Cyclin A2 wildtype (CycA2-wt), HA-Cyclin A2 hydrophobic patch mutant (CycA2-hpm) or HA-Cyclin B1 (CycB1). The input lysates and the pulled down material were analyzed by immunoblotting (IB) for the presence of pUL21a and HA-tagged cyclins. (E) Cyclin A2-associated kinase activity was immunoprecipitated from HEK293 lysates and incubated with the substrate protein pp150 and radioactively labeled γ-P32-ATP. Where indicated, equal amounts of pUL21a-wt or pUL21-RXL2mut were added to the reaction, as controlled by Coomassie staining (lower panel, IgG-LC: Immunoglobulin G light chain). The phosphorylation products were analyzed by autoradiography (upper panel).

To identify potential HCMV gene products with Cyclin A2 inhibitory function we extended our previous search for Cyclin A2-interaction motifs in virion components [53] to the whole virus proteome [56]. We found that the small HCMV early-late protein pUL21a contains two promising candidate motifs, resembling already validated RXL/Cy sequences of human Cyclin A2-CDK substrates and inhibitors (Fig. 1A). In particular, both candidate motifs possess a bulky hydrophobic residue at either position +1 or +2 relative to the RXL core. This feature is important for docking to the hydrophobic patch region in Cyclin A2 [57] and therefore can serve as a suitable criterion for the identification of candidate RXL/Cy motifs.

To test if pUL21a can physically interact with Cyclin A2 we performed a pull-down experiment, employing His-purified pUL21a as bait. We found that pUL21a-WT was able to precipitate Cyclin A2 from cell extracts with similar efficiency as the viral Cyclin A2 substrate pp150 (Fig. 1C). Pull-down of an RXL-binding deficient Cyclin A2 hydrophobic patch mutant [58] occurred only at background levels, consistent with a pUL21a-RXL-dependent recruitment of Cyclin A2. In fact, point mutation (RXL^AXA^) of the candidate motif RRLFQ (RXL2) abolished pUL21a-Cyclin A2 binding, whereas the other candidate motif, RRLAF (RXL1), was dispensable for this interaction (Fig. 1C). Cyclin B1, which binds inefficiently to RXL/Cy motifs [59], failed to co-precipitate with pUL21a (Fig. 1D), further demonstrating the specificity of pUL21a-Cyclin A2 binding.

PUL21a lacks full consensus (S/T-P-X-K/R) CDK phosphorylation sites and contains only one minimal consensus site (S/T-P) for CDK and mitogen-activated protein kinases (MAPK)-dependent phosphorylation near the N-terminus (Fig. 1B). Thus, it resembles Cyclin-CDK inhibitors (p21, p27, p57) rather than substrates. To investigate whether pUL21a functions as a Cyclin A2-CDK substrate or inhibitor *in vitro*, we included the protein in Cyclin A2 kinase assays, using pp150 as a substrate. We found that pUL21a severely compromised Cyclin A2-dependent phosphorylation of pp150 but was itself only weakly phosphorylated. In accordance with the pull-down experiment, the pUL21a-RXL2 mutant was completely inert to Cyclin A2-CDK activity (Fig. 1E). Thus, pUL21a only qualifies as a poor substrate but instead as a strong competitive inhibitor of Cyclin A2-dependent phosphorylation *in vitro*.

**Figure 2:**
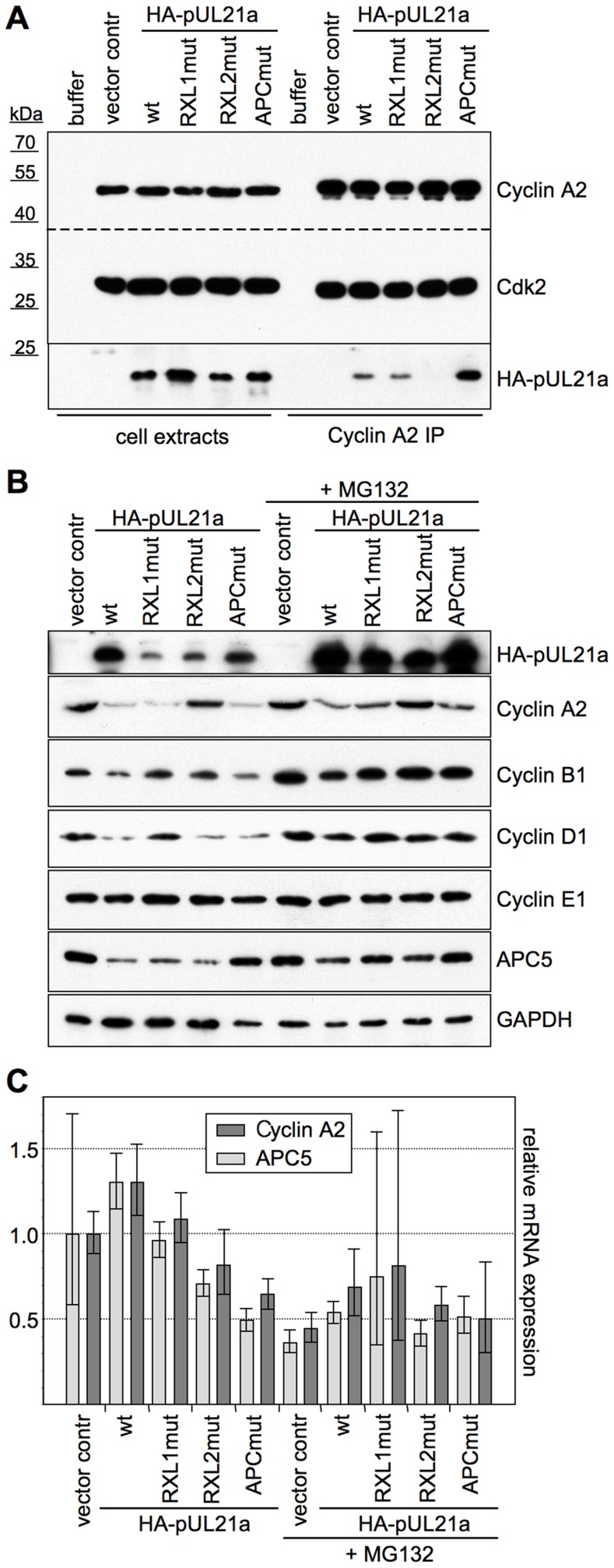
Interaction with pUL21a leads to Cyclin A2 protein degradation. HEK293 cells were transfected with the indicated pUL21a-wt and mutant expression plasmids (pUL21a-PR^AA^: APCmut) or an empty vector control. Where indicated, MG132 was added to the cells at 24 h post transfection. Cells were harvested at 48 h post transfection and processed for the following assays. (A) Cyclin A2-CDK complexes were immunoprecipitated (IP) from extracts of transfected, MG132-treated cells and analyzed by immunoblotting for the presence of Cyclin A2, CDK2 and HA-pUL21a. (B) Whole cell lysates of transfected cells were analyzed by immunoblotting for expression of the indicated proteins. (C) Total RNA preparations of transfected cells were analyzed by quantitative RT-PCR for Cyclin A2 and APC5 mRNA expression. Error bars indicate the standard deviations of triplicate samples.

In order to validate the pUL21a-Cyclin A2 interaction under more physiological conditions, we aimed to co-immunoprecipitate both proteins from pUL21a-transfected human cells (Fig. 2A). To improve detection of pUL21a, we stabilized the protein by MG132-mediated inhibition of the proteasome. Under these conditions, we were able to co-precipitate a significant fraction of pUL21a with endogenous Cyclin A2-CDK2 complexes. Like in the preceding experiments, an intact RXL2 motif was required for this interaction. The amount of Cyclin A2-bound pUL21a was increased when using a previously described pUL21a-PR^AA^ mutant (APCmut), deficient in APC/C binding [42]. This was most probably due to the higher availability of pUL21a-APCmut and suggests that Cyclin A2-CDK binding to pUL21a was not saturated.

When comparing cyclin protein levels of MG132-treated and untreated cells, it became apparent that in the presence of an active proteasome, pUL21a transfection resulted in a marked, RXL2-dependent decrease of Cyclin A2 and an RXL1-dependent decrease of Cyclin D1 (Fig. 2B). These effects were specific since Cyclins B1 and E1 were unaffected, and they were similar in strength as the APC/C binding site-dependent down-regulation of APC5 [42]. Furthermore, both Cyclin A2 and APC5 mRNA levels were not influenced by pUL21a (Fig. 2C). Thus, destabilisation of the Cyclin A2 protein appears to be the primary function of pUL21a-Cyclin A2 binding *in vivo*. In addition, the RXL1-dependent down-regulation of Cyclin D1 pointed to a distinct function of the other putative cyclin docking motif.

**Figure 3:**
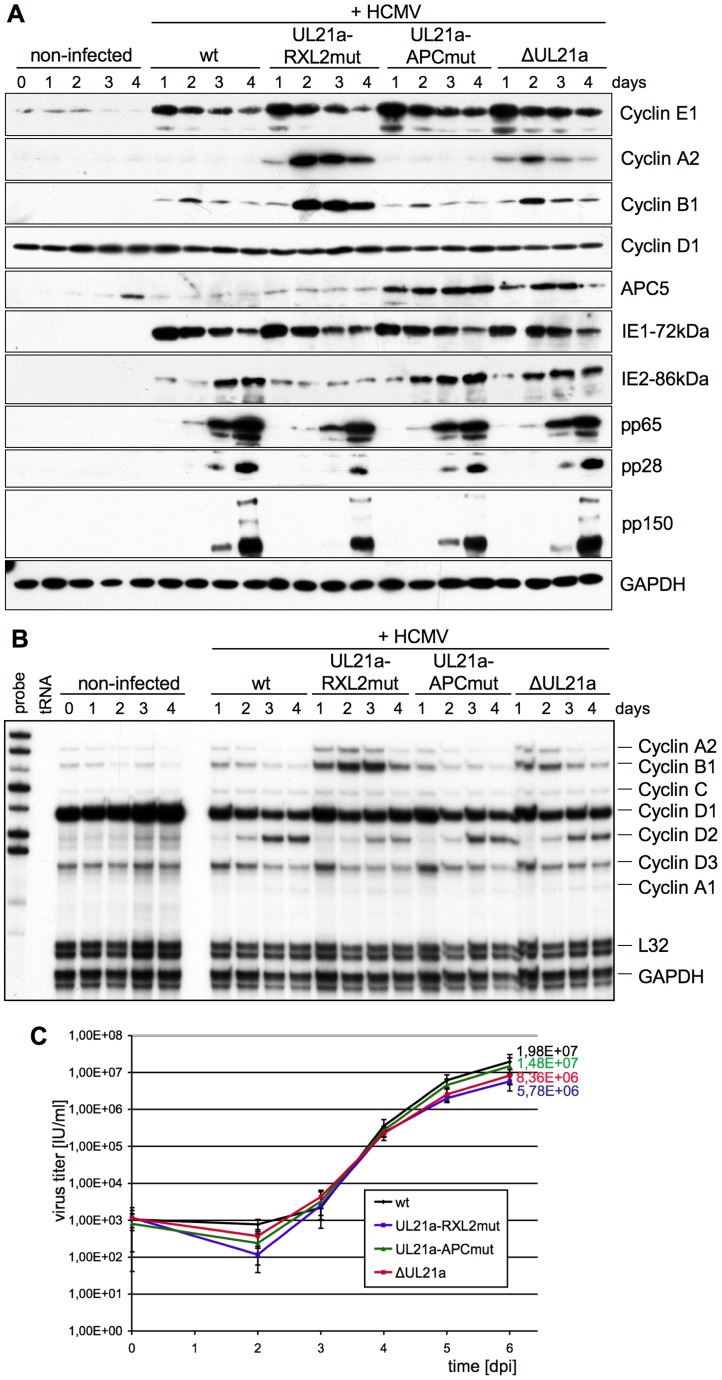
The pUL21a-RXL/Cy motif is required for Cyclin A2 repression by HCMV. Density arrested human embryonic lung (HEL) fibroblasts were infected with HCMV reconstituted from TB40-BAC4-wt or derivatives carrying the indicated UL21a mutations. Cells were harvested at regular intervals and analyzed for viral and cellular gene expression by immunoblotting (A) and ribonuclease protection assay (B). Cell culture supernatants were analyzed for the number of IE protein-forming units (IU) by virus titration (C). Results are represented as mean values plus standard deviations of triplicate samples.

We then set out to investigate the functional consequences of pUL21a-Cyclin A2 interaction in the context of viral infection. To this end we introduced the RXL2 mutation into the HCMV genome by traceless BAC-mutagenesis and analyzed the recombinant virus in direct comparison to WT, UL21a deletion (ΔUL21a) and UL21a-PR^AA^ mutant (APCmut) viruses. To ensure a synchronous start of lytic gene expression in infected cells, we used a high multiplicity of infection (MOI) and fibroblasts that were arrested in G0/G1 by contact inhibition. Accordingly, except the G1-specific Cyclin D1, cell cycle factors were barely detectable at the time of infection (Fig. 3A). Whereas Cyclin D1 expression was largely unaffected by HCMV infection and all four viruses led to a strong and sustained induction of Cyclin E1, they markedly differed with respect to Cyclin A2, Cyclin B1 and APC5 protein regulation. Both UL21a deletion and RXL2 point mutation abolished the characteristic block of Cyclin A2 expression observed in HCMV-WT infected cells. Whereas Cyclin A2 mRNA levels were only slightly elevated (see Fig. 3B), the effect was most pronounced at the level of protein expression, consistent with the observed proteasome dependency of Cyclin A2 down-regulation in pUL21a-transfected cells (Fig. 2). Remarkably, the RXL2 point mutation resulted in considerably higher peak levels of Cyclin A2 protein expression than the deletion of the whole UL21a open reading frame. This may be explained by the fact that the APC/C inhibitory function of UL21a is not affected by the loss of Cyclin A2 binding (see APC5 levels in Fig. 3A) and thus can contribute to Cyclin A2 stabilization. Cyclin A2 induction in HCMV-UL21a-RXL2mut-infected cells was followed by a strong up-regulation of Cyclin B1 mRNA and protein expression, most likely due to Cyclin A2-dependent transcriptional activation of the Cyclin B1 promoter [23]. Again, the effects of UL21a deletion were much weaker, hardly exceeding the moderate levels of Cyclin B1 induction in HCMV-WT-infected cells. Thus, the simultaneous loss of Cyclin A2 and APC/C-binding sites in pUL21a masks the full potential of its Cyclin A2 and B1-directed negative control function.

We next asked what impact, if any, the increased amounts of Cyclin A2 and B1 at early-late times of infection have on viral replication. We found that the UL21a-RXL2 mutation caused a clear delay of up to 24 h in the overall accumulation of late (pp28, pp150) gene products. Furthermore, the characteristic late increase in IE2 protein expression was also suppressed in HCMV-UL21a-RXL2mut-infected cells (Fig. 3A). This correlated with an almost fourfold reduction in viral progeny production compared to HCMV-WT (Fig. 3C).

**Figure 4:**
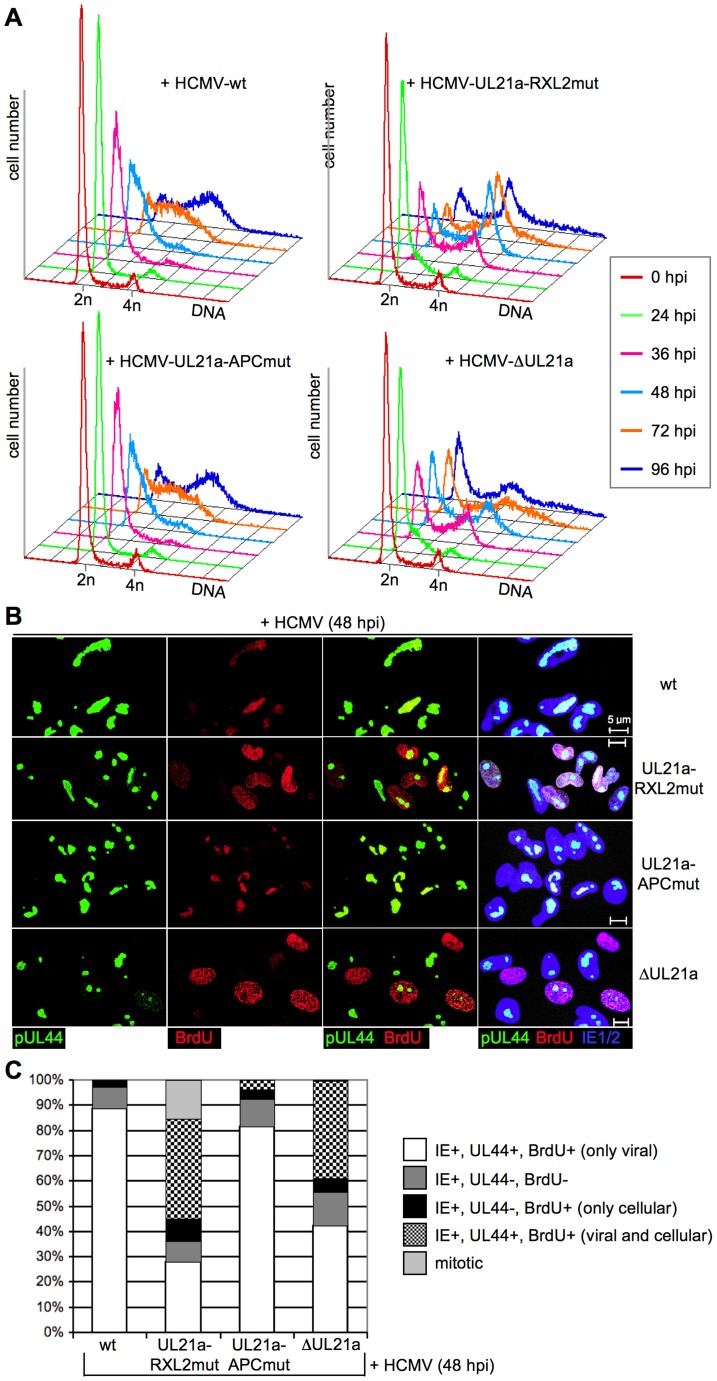
Cyclin A2 expression relieves the block of cellular DNA synthesis in HCMV-infected cells. (A) Cells were infected as described in the legend to figure 3. Cell cycle progression was monitored by flow cytometric analysis of cellular DNA content (n =  haploid number of chromosomes). (B) To distinguish cellular from viral DNA replication, infected cells were labeled with BrdU at 48 h post infection (hpi). The sites of BrdU incorporation were stained by immunofluorescence and compared with the localization of pUL44-containing viral replication compartments, using confocal microscopy. In the right column, merged images of the middle columns are shown. (C) Quantitative analysis of cells undergoing cellular and/or viral DNA replication at 48 hpi. Mitotic cells were identified by DAPI co-staining. The results represent at least 200 randomly selected IE1/2-positive cells.

The changes induced by the loss of pUL21a-mediated control over Cyclin A2 became more evident when the cell cycle profiles of infected cells were analyzed. Between 24 and 48 h post infection, both UL21a deletion and RXL2 mutation caused a rapid increase in the rate of DNA synthesis, leading to an accumulation of cells with 4n DNA content (Fig. 4A). This increase was due to cellular rather than viral DNA replication, as indicated by BrdU pulse-labeling experiments. Both in HCMV-WT and HCMV-APCmut-infected cells the sites of BrdU incorporation co-localized with pUL44, which is the processivity factor of the viral DNA polymerase and a well-accepted marker of ongoing viral DNA replication [60], [61]. This restriction of DNA synthesis to viral replication compartments was overcome in the majority of HCMV-ΔUL21a and HCMV-RXL2mut-infected cells. There, nucleotide incorporation was detected throughout the nucleus, both in pUL44-positive and negative regions (Fig 4B–C). The presence of cellular DNA synthesis in these cells was confirmed by suppressing viral replication with foscarnet (PFA), a selective inhibitor of herpesviral DNA polymerases. Although PFA treatment, consistent with published data [62], caused some delay in S-phase progression, a large number of HCMV-ΔUL21a and HCMV-UL21a-RXL2mut-infected cells had doubled their DNA content after 96 h (Fig. S1). Thus, the pUL21a-Cyclin A2 interaction is essential for the block of cellular DNA synthesis in HCMV-infected cells.

**Figure 5:**
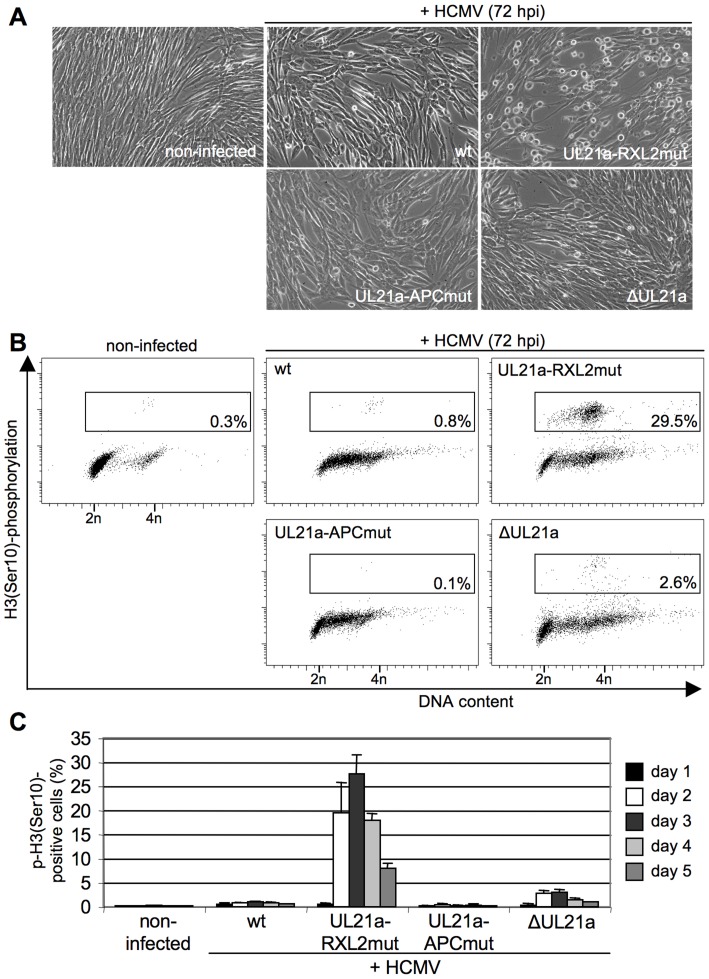
Cyclin A2 up-regulation results in mitotic entry of infected cells. Cells were infected as described in the legend to figure 3. (A) At 72 hpi, their morphology was examined by phase-contrast microscopy to detect cell rounding. (B, C) Cells were further stained with propidium iodide, IE1/IE2-and phospho-histone H3(Ser10)-specific antibodies and analyzed by flow cytometry to determine the proportion of infected cells with (pre-)mitotic chromosome condensation. The results of one representative experiment (A, B) and of five independent experiments (C) are shown.

We then examined whether the Cyclin A2-induced progression through S-phase is followed by mitotic entry of infected cells. Entry into mitosis is marked by cell rounding and chromatin condensation and therefore can be readily visualized by phase contrast microscopy and DAPI staining. Both methods revealed a high number of mitotic cells at 48 h after infection with the UL21a-RXL2 mutant virus (Fig. 4C, 5A). To quantitatively assess the number of mitotic cells, we performed flow cytometry of histone H3-serine 10 phosphorylation (pH3ser10), a marker of condensed chromatin. Parallel analysis of DNA content and IE1/2 protein expression in combination with an appropriate gating strategy ensured that only single, IE-positive cells were evaluated (Fig. S2). We found that the percentage of HCMV-UL21a-RXL2mut-infected cells displaying H3(ser10) phosphorylation increased from about 20% at day 2 to almost 30% at day 3 before it declined again to just under 10% at day 5 (Fig. 5B–C). Some cells had undergone chromatin condensation prematurely, i.e. before completion of DNA replication (Fig. 5B). In case of UL21a deletion, the proportion of mitotic cells was also increased compared to HCMV-WT, but remained constantly below 3% and showed no signs of premature mitotic entry. This low rate of mitosis is well supported by the only moderate strength of Cyclin B1 induction in these cells (Fig. 3A). As it is rather unusual that a point mutation has a stronger phenotype than a whole gene knockout, it was important to ensure that cyclin up-regulation and mitotic entry were not caused by unwanted secondary mutations in the genome of the UL21a-RXL2-mutant virus. We therefore repaired the RXL2 mutation and analyzed the cell cycle effects of the UL21a-RXL2-revertant virus. Cyclin A2 and B1 protein expression as well as mitotic cell numbers were reverted to HCMV-WT levels (Fig. S3), demonstrating that the observed changes are specifically linked to the pUL21a-RXL2 motif. We concluded that pUL21a-Cyclin A2 interaction is not only required for the inhibition DNA synthesis in HCMV-infected cells but also for the inhibition of mitotic entry.

It is important to note that even in case of the RXL2 mutant the majority of infected, Cyclin A2 expressing cells did not traverse the G2/M boundary but remained in S/G2. In contrast to the mitotic cell population, these pH3(ser10)-negative cells appeared to support viral DNA replication to a reasonable extent as many of them acquired a greater than 4n DNA content in a PFA-sensitive manner (Fig. 5B, Fig. S1). This may explain why Cyclin A2 up-regulation has no stronger effect on virus growth (Fig. 3C).

To test whether the observed effects of UL21a-RXL2 mutation on cell cycle progression and virus replication are a TB40-specific phenomenon, we introduced the same mutation in the highly fibroblast-adapted HCMV strain AD169. Following infection of confluent fibroblasts, the AD169-UL21a-RXL2 mutant virus induced similar levels of mitotic entry (Fig. S4A) as the TB40 counterpart (Fig. 5A, Fig. S3B) and also had a similarly moderate growth defect (Fig. S4B). These results argue against a virus strain dependency of the UL21a-RXL2mut phenotype.

**Figure 6:**
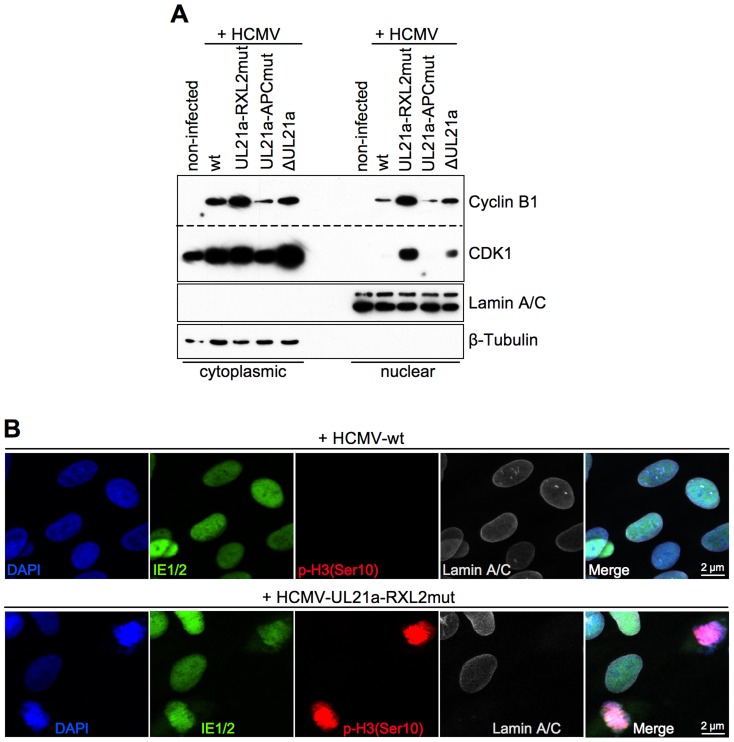
Disruption of pUL21a-Cyclin A2 interaction facilitates nuclear translocation of Cyclin B1-CDK1 and nuclear envelope breakdown in HCMV-infected cells. Cells were infected as described in the legend to figure 3. (A) At 48 hpi, nuclear and cytoplasmic fractions were prepared and analyzed for the presence of Cyclin B1 and CDK1 protein. The purity of both fractions was controlled by immunoblot detection of Lamin A/C and β-Tubulin. (B) In parallel, cells were analyzed by immunofluorescence microscopy for markers of chromosome condensation (histone H3 phosphorylation at serine 10) and nuclear envelope integrity (Lamin A/C). Shown are representative images. All cells were confirmed to be HCMV-positive by IE1/IE2 antibody co-staining.

Under normal circumstances, the duration of mitosis in cultured fibroblasts is about 1 h. The finding of HCMV-UL21a-RXL2mut-infected cells remaining at high numbers in mitosis over days pointed to a blockade or at least a severe prolongation of the mitotic process. We reasoned that the previously reported restriction of Cyclin B1-CDK1 activity to the cytoplasm of HCMV-infected cells [47] might prevent the nuclear envelope breakdown in late prophase [63]. To test this possibility, we analyzed the nucleo-cytoplasmic distribution of Cyclin B1-CDK1 and the integrity of the nuclear lamina. As expected, we were able to confirm the cytoplasmic sequestration of CDK1 by HCMV-WT. However, we observed a high proportion of CDK1 in the nuclear fraction of UL21a-RXL2mut-infected cells (Fig. 6A), suggesting that Cyclin A2 down-regulation by pUL21a is also responsible for the limited nuclear availability of this essential mitotic kinase during HCMV infection [47]. Interestingly, when we checked the nucleo-cytoplasmic distribution of Cyclin A2 and pUL21a (Fig. S4), we found that Cyclin A2 resembled CDK1 in a way that nuclear translocation was only possible in the absence of pUL21a binding. In contrast, pUL21a itself was found in both cellular compartments. In accordance with the observed nuclear entry of Cyclin A2, Cyclin B1 and CDK1, chromosome condensation in mitotic HCMV-UL21a-RXL2mut-infected cells was accompanied by a loss of the nuclear envelope (Fig. 6B). Hence, there was no evidence for a block of prophase-prometaphase transition.

**Figure 7:**
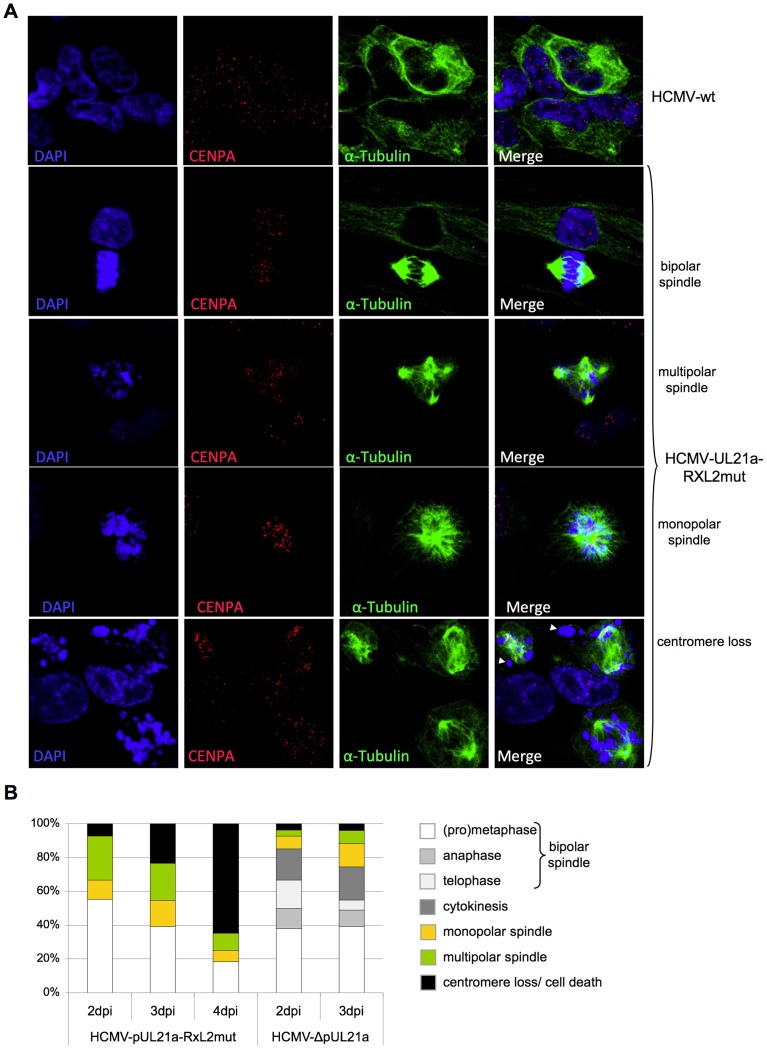
Lack of Cyclin A2 degradation promotes metaphase arrest, aberrant spindle formation and chromosomal instability in HCMV-infected cells. Cells were infected with HCMV-wt, HCMV-UL21a-RXL2mut or HCMV-ΔUL21a. Infected cells were examined by immunofluorescence microscopy for localization and structural organization of chromosomal material (DAPI staining), centromeres (CENP-A staining) and mitotic spindles (α-Tubulin). Representative images are shown in (A). Chromosomes lacking centromeres and accordingly have lost spindle attachment are marked by arrowheads. Quantitative data based on at least 100 mitotic cells per sample are presented in (B). Non-mitotic cells were not included in this quantitative analysis.

In order to identify the step in mitosis that is impeded in cells infected with the UL21a-RXL2 mutant, we next visualized mitotic figures by fluorescent staining of chromosomes (DAPI), centromeres (CENP-A) and the spindle apparatus (α-Tubulin). The results were clear-cut. At 2 days post infection, the large majority of mitotic cells had accumulated in metaphase whilst anaphase and telophase figures were completely absent (Fig. 7A–B). Since in HCMV-ΔUL21a-infected cells all mitotic stages were represented, this metaphase arrest appeared to be a direct consequence of the still intact APC/C-inhibitory function in pUL21a-RXL2mut. Remarkably, not all metaphase cells contained a regular, bipolar mitotic spindle. About one third displayed an aberrant, mono- or multipolar spindle formation (Fig. 7A–B). This phenomenon was not specific for the UL21a-RXL2 mutant as it was also observed in case of the UL21a deletion virus (Fig. 7B). Over time, more and more of the metaphase-arrested cells acquired additional pathological features and ultimately disintegrated entirely. The most obvious alteration was the appearance of dispersed chromosomal material that no longer co-localized with the centromere-specific histone H3 variant CENP-A (Fig. 7A–B). Whereas the CENP-A-containing foci were still in contact with the mitotic spindle, the respective chromosomal material was detached (Fig. 7A). This indicates that these chromosomes had lost their centromeric region, most likely as a result of chromosomal instability. The centromere loss occurred in HCMV-infected cells as demonstrated by IE1/IE2-costaining (Fig. S6). Thus, a decomposition of chromosomes appeared to characterize the end-stage of the aberrant mitotic process induced by UL21a-RXL2-deficient HCMV.

**Figure 8:**
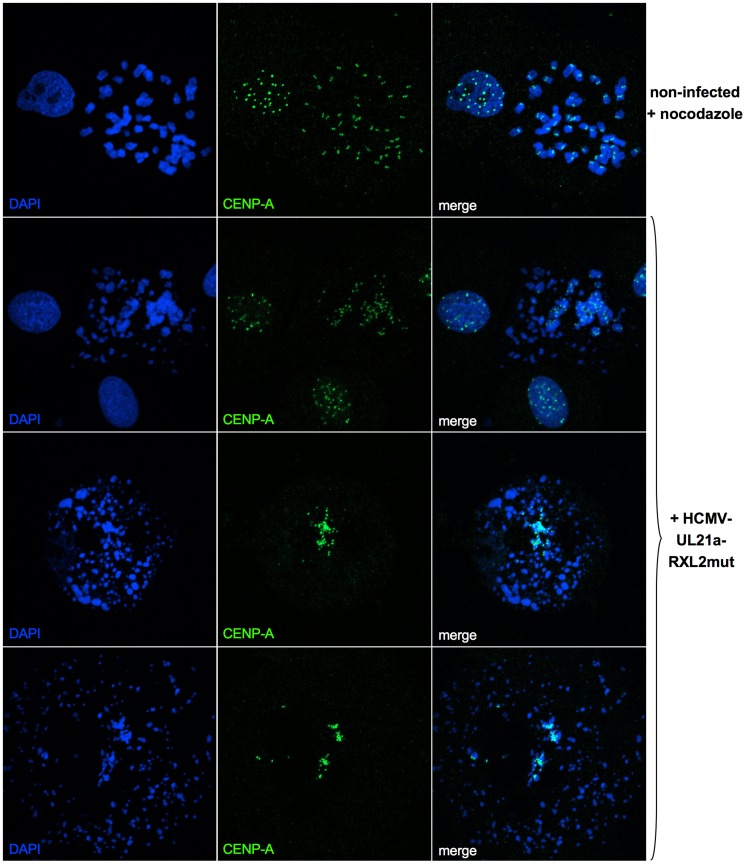
Generalized chromosome shattering in HCMV-UL21a-RXL2mut-infected cells. Metaphase spreads from nocodazole-treated, non-infected cells were subjected to CENP-A immunofluorescence and DAPI staining and compared to equally prepared material of cells 3 days post HCMV-UL21a-RXL2mut-infection. Representative images are shown.

To examine the structural integrity of mitotic chromosomes in more detail, we analyzed metaphase spreads of HCMV-UL21a-RXL2mut-infected cells by various methods. First, we re-applied CENP-A and DAPI co-staining to confirm the assumed chromosomal disintegration. Numerous acentric chromatid fragments were found dispersed to the periphery of the spreads, whereas the residual, CENP-A-positive material often clustered in central, metaphase plate-like structures (Fig. 8). Non-infected, prometaphase-arrested control cells, in contrast, showed regular pairs of sister chromatids and centromeres (Fig. 8, upper panel). The sheer extent of chromosome shattering in many HCMV-UL21a-RXL2mut-infected cells (Fig. 8, lower panels) pointed towards a general, and not a chromosome-specific destruction process.

**Figure 9:**
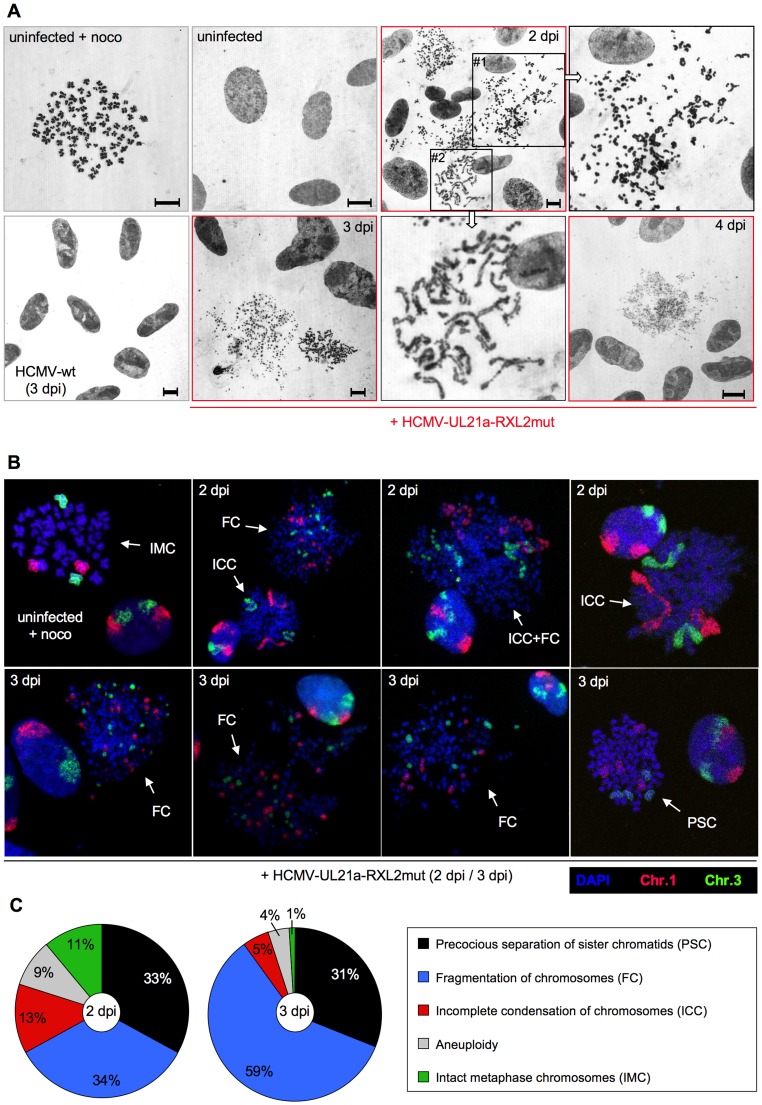
Precocious separation of sister chromatids and progressive chromosome fragmentation predominate the chromosomal appearance of HCMV-UL21a-RXL2mut-infected cells. (A) Metaphase spreads from nocodazole-treated, non-infected cells were subjected to Giemsa staining and compared to equally prepared chromosomal material of HCMV-wt and HCMV-UL21a-RXL2mut-infected cells at 2 to 4 days post infection (dpi). Where indicated, magnified views of the encircled areas #1 and #2 in the adjacent image of RXL2mut-infected cells are shown. Scale bars: 5 µm. (B) Metaphase spreads were analyzed by fluorescence in situ hybridization (FISH) using whole chromosome painting probes for chromosomes 1 and 3. DNA was counterstained with DAPI. Typical examples of cells with intact metaphase chromosomes (IMC), fragmented chromosomes (FC), incomplete chromosome condensation (ICC) or precocious separation of sister chromatids (PSC) are shown and labeled accordingly. (C) Quantitative evaluation of FISH analysis based on at least 100 mitotic HCMV-UL21a-RXL2mut-infected cells per sample. Non-mitotic cells were not included in the analysis.

Giemsa staining revealed further chromosomal abnormalities, contrasting with the characteristic X-shaped chromosomal appearance of prometaphase-arrested control cells (Fig. 9A, upper left panel). Besides chromosomal fragmentation, HCMV-UL21a-RXL2mut-infected, mitotic cells displayed incompletely condensed chromosomes (Fig. 9A, magnified view #2) or showed signs of precocious separation of sister chromatids (Fig. 9A, magnified view #1). In addition, the extent of chromosomal fragmentation increased over time and “pulverized” chromosomes dominated the picture of mitotic cells at 3–4 days post infection (Fig. 9A, lower panel). Notably, the remaining non-mitotic HCMV-UL21a-RXL2mut-infected cells appeared at this time with fully developed viral replication compartments, visible as dark-stained intranuclear regions. This is in accordance with the observation of viral DNA replication in the G2-arrested, non-mitotic subpopulation (Fig. 5B, Fig. S1) and with the only moderate growth defect of pUL21a-Cyclin A2 binding-deficient viruses (Fig. 3C). It appears that not Cyclin A2 expression per se but mitotic entry had deleterious consequences for HCMV-infected cells.

Chromosome fragmentation is often difficult to distinguish morphologically from premature, incomplete chromosome condensation [64]. To obtain further proof for the presence of chromosomal breakage and to enable a more quantitative assessment of the different chromosomal abnormalities, we performed fluorescence in situ hybridization (FISH) analysis of two large chromosomes, 1 and 3 (Fig. 9B–C). Incompletely condensed chromosomes were recognized by their extended conformation or beads-on-a-string appearance. A clear indication of chromosome breakage was the random distribution of chromosome 1 or 3 fragments over the whole metaphase spread. The whole chromosome FISH analysis confirmed the presence of both phenotypes. At 2 days post infection, some HCMV-UL21a-RXL2mut-infected mitotic cells even showed a mixed phenotype with features of both prematurely condensed and partially fragmented chromosomes (Fig. 9B), suggesting that in these cells premature condensation gives way to chromosomal breakage [65]. In support of this view, the percentage of mitotic cells with fragmented chromosomes almost doubled between 2 and 3 days post infection (from 34% to 59%), at the expense of cells with incompletely condensed and normal metaphase chromosomes (Fig. 9C). Notably, about one third of mitotic cells showed no major signs of chromosomal damage at day 2 to 3 post HCMV-UL21a-RXL2mut infection but instead contained precociously separated sister chromatids (Fig. 9B–C). This points to a phenomenon called cohesion fatigue, which is often found in cells arrested or delayed at metaphase [66].

**Figure 10:**
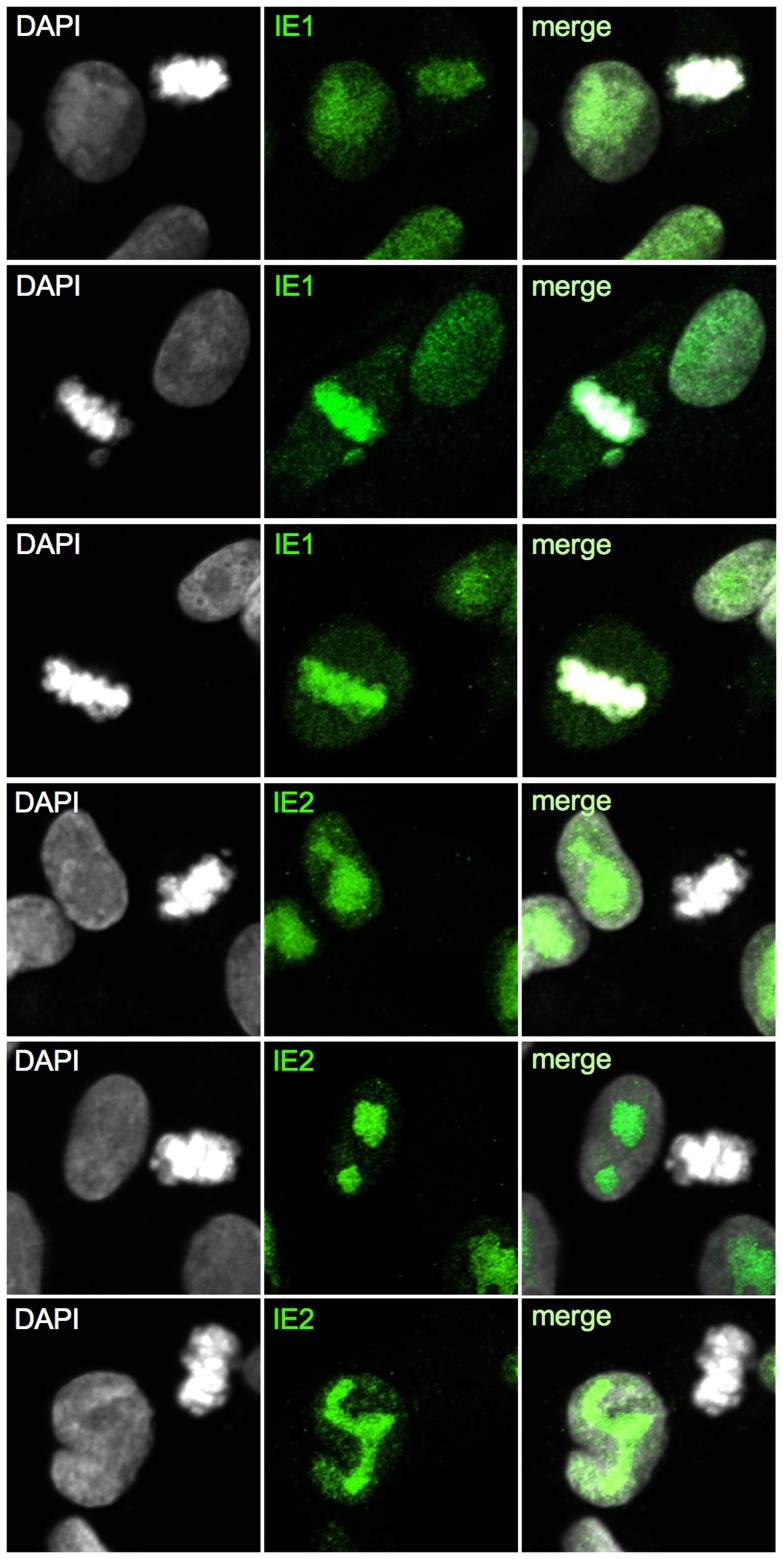
Selective down-regulation of IE2 protein in mitotic cells. Cells were infected with HCMV-UL21a-RXL2mut and analyzed at 72 hpi by confocal immunofluorescence microscopy for the expression and subcellular localization of IE1 and IE2 proteins. DAPI staining enabled discrimination of mitotic from non-mitotic cells. Representative images are shown. The right column displays the merged fluorescence channels.

**Figure 11:**
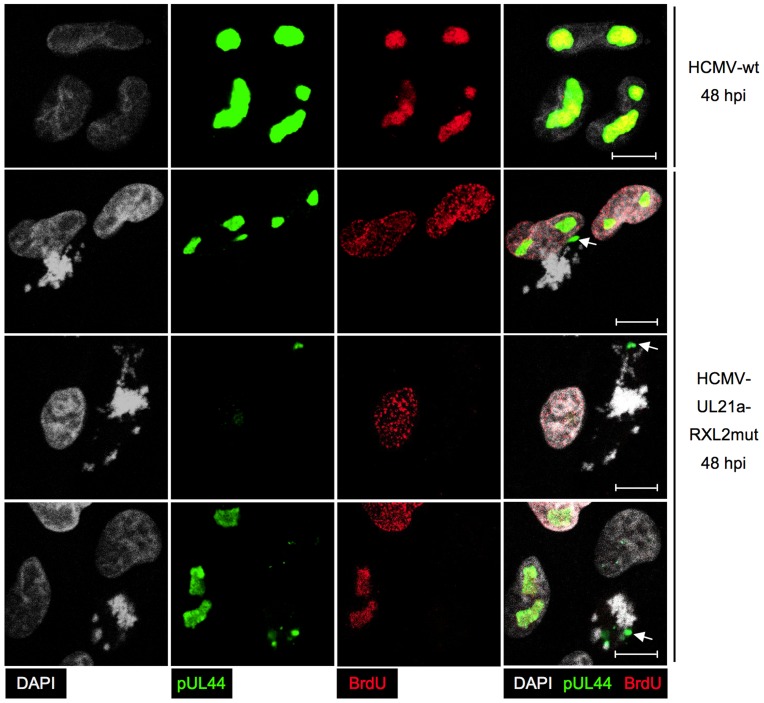
Impaired viral DNA replication in mitotic cells. Cells were infected with HCMV-UL21a-RXL2mut and prepared at 48 hpi for immunofluorescence microscopy. Chromatin condensation (DAPI), localization of viral DNA replication compartments (pUL44) and DNA synthesis (BrdU) were analyzed and representative images are shown. The right column displays the merged fluorescence channels. Arrows point to the small pUL44-positive areas in metaphase cells. Scale bars: 5 µm.

Having completed the initial characterization of virus-induced mitotic aberrations, we were next interested to gain insight into the implications of mitotic entry for HCMV replication. First, we analyzed the consequences for major immediate early proteins IE1 and IE2 (Fig. 10). Both nucleoproteins were easily detectable in the non-mitotic fraction of HCMV-UL21a-RXL2mut-infected cells and showed a regular nuclear localization pattern, with IE1 being evenly distributed and IE2 accumulating in viral replication compartments [67]. In mitotic cells, however, the picture was dramatically different. Whereas IE1 was found associated with metaphase chromosomes, as expected from published work [68], [69], IE2 protein expression was below detection limit (Fig. 10). The lack of this essential viral transcription factor suggests that virus replication is aborted in mitosis. This was further supported when we examined the morphology and activity of viral replication compartments in metaphase-arrested cells (Fig. 11). Judged on the basis of pUL44 staining, the replication compartments appeared much smaller compared to interphase cells and were displaced to the cellular periphery. Moreover, no incorporation of BrdU was detectable suggesting that viral DNA synthesis and accordingly progression of the infectious cycle was stalled. We concluded that the pUL21a-dependent arrest at the G1/S-transition eventually protects HCMV-infected cells from entering an abortive mitotic stage in which the virus can neither replicate nor enable the infected cell to exit from.

## Discussion

**Figure 12:**
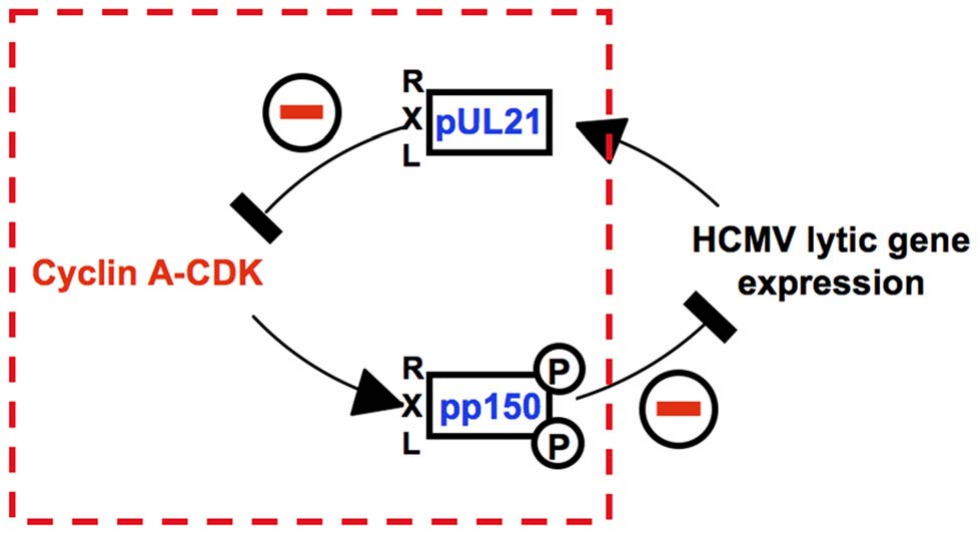
An RXL-based molecular interface between HCMV and Cyclin A2. The interface consists of two classical Cyclin A2 interaction motifs that have been acquired by viral gene products pp150 and pUL21a. The tegument protein pp150 is a substrate of Cyclin A2-dependent phosphorylation and blocks viral gene expression when Cyclin A2-CDK activity is high. Once viral gene expression has started, the early-late protein pUL21a blocks Cyclin A2-CDK activity by Cyclin A2 degradation.

The antagonism between Cyclin A2-CDK activity and viral gene expression has been recognized as a characteristic hallmark of HCMV-cell cycle interactions [7], [46], [51], [52]. Recently, we discovered that HCMV employs an RXL/Cy motif in the pp150 tegument protein to sense the cellular Cyclin A2 status right at the beginning of an infection [51], [53]. This sensing mechanism is required to restrict the onset of IE gene expression to the G0/G1 phase, where Cyclin A2-CDK is inactive. Here, we found how HCMV makes use of another RXL/Cy motif in the early gene product pUL21a to maintain the status of low Cyclin A2-CDK activity after lytic gene expression has started. This mechanism is not only required to block cellular DNA synthesis but ensures that viral replication is not abrogated by entry into mitosis. Possibly, it also protects pp150 at the stage of virion assembly from Cyclin A2-CDK-dependent phosphorylation so that pp150 can support the onset of lytic gene expression in newly infected G0/G1 cells. Such cooperative action would explain why the progeny of UL21a-deficient HCMV has a reduced infectivity despite pUL21a itself is not a component of viral particles [55]. Thus, we suggest that both virus-encoded interactors of Cyclin A2 constitute a control circuitry (Fig. 12) that synchronizes the HCMV lytic cycle to the cell division cycle in a way that secures efficient viral growth.

**Figure 13:**
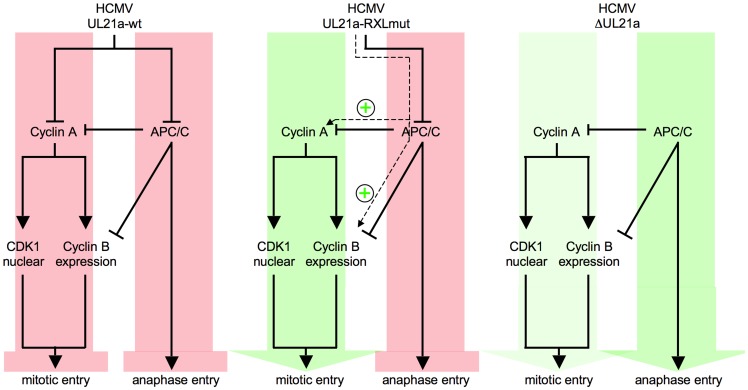
Crosstalk and antagonism between Cyclin A2 and APC/C-inhibitory functions of HCMV-pUL21a. The UL21a gene product conveys to independent cell cycle activities, inhibition of Cyclin A2-dependent protein phosphorylation and inhibition of APC/C-dependent protein ubiquitination. Cyclin A2 inhibition, by impinging on Cyclin B1 accumulation and CDK1 nuclear translocation, prevents HCMV-infected cells from entering mitosis. APC/C inhibition leads to a block of metaphase-anaphase transition (left panel). If the Cyclin A2-inhibitory function is selectively impaired by UL21a-RXL mutation, the remaining APC/C-inhibitory function favors the accumulation of Cyclin A2, Cyclin B1, nuclear CDK1 and hence mitotic entry, while anaphase entry is still blocked (middle panel). If both functions are ablated by UL21a knockout, mitotic entry is still possible but much less efficient. Once in mitosis, the HCMV-ΔUL21a-infected cells can proceed to ana- and telophase. Green or red arrows represent the net effects of the indicated HCMV-UL21a variants on G2/M and metaphase/anaphase transitions.

Cyclin A2 destruction is after APC4/5 destruction [42] the second cell cycle regulatory function ascribed to pUL21a. This small protein appears to be perfectly designed to confer its own instability to every protein it binds to. Importantly, we found (Fig. 3A–B, 6A) that Cyclin A2 down-regulation by pUL21a also accounts for the only moderate expression of Cyclin B1 and the previously reported cytoplasmic sequestration of CDK1 in HCMV-infected cells [47]. Because the APC/C ubiquitin ligase is required for proteasomal degradation of Cyclin A2 and Cyclin B1 from M to G1 phase [13], the two functions residing in pUL21a, APC/C inhibition and Cyclin A2 degradation, inversely regulate Cyclin A2 and Cyclin B1 abundance (Fig. 13). This leads to the seemingly paradoxical situation that pUL21a-RXL point mutation has a much stronger effect on Cyclin A2 and B1 expression (Fig. 3A–B), CDK1 nuclear translocation (Fig. 6A) and mitotic entry (Fig. 5) of infected cells than deletion of the whole UL21a open reading frame (Fig. 13). Why has HCMV concentrated two potent antagonistic cell cycle activities on the same protein? Probably, HCMV benefits from stabilization of the many other APC/C substrates [44], [70] and, by integrating a Cyclin A2-binding motif into the same regulatory module, has found an elegant way to counteract the negative consequences of increased Cyclin A2 and B1 stability. Furthermore, it might be beneficial for virus replication if the APC/C-inhibitory function of pUL21a, which alone elicits mitotic entry and fatal metaphase arrest, is balanced by the G1 arrest function of pUL21a-mediated Cyclin A2 degradation (Fig. 13). Our data do not exclude the existence of additional mechanisms contributing to Cyclin A2 down-regulation at the level of transcription [50]. In fact, repression of the Cyclin A2 promoter in the early phase of infection [46] might account for the only moderate effect of pUL21a-RXL mutation on Cyclin A2 protein expression at 24 hpi (Fig. 3A).

Although Cyclin A2 is an important regulator of S phase [71], [72], the necessity of pUL21a-Cyclin A2 interaction for the inhibition of cellular DNA synthesis in HCMV-infected cells is an unexpected result. Previous evidence suggested that HCMV interferes primarily with the process of replication licensing, by inhibiting the loading of the MCM2-7 helicase complex onto chromatin [39], [41]. Two mechanisms have been proposed: up-regulation of the cellular licensing inhibitor Geminin [39] and expression of the viral licensing inhibitor pUL117 [40]. In addition, a post-licensing inhibition of the MCM2-7 complex by direct physical interaction between the MCM3 acetylase MCM3AP and HCMV-IE2 has been reported [73]. Conversely, low Cyclin A2-CDK activity is known to promote, rather than constrain, MCM2-7 loading [74]–[76], arguing against a causative role of pUL21a-Cyclin A2 interaction in the viral inhibition of pre-replicative complex formation. Only recently, Cyclin A2-MCM7 binding was described to be critical for the S-phase promoting function of Cyclin A2 [77]. Two scenarios can be considered to explain the apparent dominance of Cyclin A2 over other virus-encoded control mechanisms: either the MCM-directed viral inhibitors alone are not sufficient to maintain a tight and stable block to the onset of cellular replication or they are negatively influenced by Cyclin A2 expression.

Notably in this context, Cyclin A2 over-expression [51] as well as UL21a deletion [78] have been shown to specifically impair mRNA expression of the essential viral trans-activator and S phase inhibitor IE2 [38], [73]. The causal chain from pUL21a via Cyclin A2 to IE2 expression and inhibition of cellular DNA synthesis was confirmed by another report by Caffarelli *et al* that appeared while this manuscript was in preparation [79]. Importantly, Caffarelli *et al* were able to overcome the negative effects of pUL21a-RXL2 mutation on IE2 expression and virus replication by Cyclin A2 knockdown [79]. In the present study, IE2 protein accumulation at late times of infection was suppressed by UL21a-RXL point mutation but only marginally affected by UL21a deletion (Fig. 3A), correlating well with their different impact on Cyclin A2 expression and mitotic entry (see above). In fact, with the elimination of IE2 protein in mitosis (Fig. 10) we discovered a further level of IE2-specific regulation, contributing to the overall decrease in IE2 expression of HCMV-UL21a-RXL2mut-infected cells. Caffarelli *et al* [79] possibly missed mitotic entry of UL21a mutant-infected cells because their cell cycle analysis was carried out at early times of infection (24–48 hpi) and in the presence of cell cycle-retarding concentrations of phosphonoacetic acid [51]. An exact side-by-side comparison, however, of Cyclin A2-mediated effects on IE2 and the cell cycle in this and previous studies is difficult, given that all previous analyses employed the highly laboratory-adapted strain AD169 [51], and used either proliferating [78], [79] or Cyclin A2-overexpressing cells [51] for infection. Here, in contrast, the low-passage endotheliotropic strain TB40 and growth-arrested cells were used as starting materials for most experiments. Given that in our cellular system an AD169-UL21a-RXL2 mutant showed a very similar cell cycle and virus growth phenotype (Fig. S4) as the corresponding TB40 mutant (Fig. 3C, Fig. 5A), it appears that different host cell conditions at the time of infection are the most likely explanation for the more severe growth defects reported for UL21a-RXL and deletion mutants in the AD169 background [78], [79]. Although we provided clear evidence for an abrogation of viral replication after entry into mitosis (Fig. 10–11), it appears that the remaining two thirds of cells in interphase (mainly G2 phase, see Fig. 4–5, Fig. S1) supported viral DNA replication and release of HCMV progeny to a reasonable extent. It remains to be determined how distinct HCMV genotypes and varying infection conditions influence the actual outcome of pUL21a-Cyclin A2 interaction on IE2 expression and virus growth.

Animal CMVs lack a Cyclin A2-binding site in their pp150 homologues and accordingly can initiate viral gene expression independent of the host cell cycle state [53]. Except for primate CMVs, where the Cyclin A2-destabilizing function of pUL21a is conserved [79], animal CMVs lack also a pUL21a homologue and therefore, not surprisingly, murine CMV (MCMV) has been found to induce not only Cyclin E1 but also Cyclin A2-dependent kinase activity [51]. However, Cyclin A2 up-regulation does not result in mitotic entry of MCMV-infected cells, which instead become arrested in G1 and G2 by a yet unknown IE3-dependent mechanism [80]. Both G1 and G2-arrested cells support MCMV DNA replication with similar efficiency [80]. This resembles the situation in HCMV-UL21a-RXL2mut and HCMV-ΔUL21-infected G2 cells where PFA-sensitive viral DNA synthesis leads to a greater than 4n DNA content (Fig. 5B, Fig. S1). Thus, it appears that animal CMVs have evolved their own, pUL21a-independent cell cycle arrest mechanisms to prevent mitotic entry, thereby counteracting the negative consequences that Cyclin A2 up-regulation can have for viral replication.

The removal of pUL21a-dependent Cyclin A2 repression reveals the full mitogenic potential of HCMV. The UL21a-RXL-mutant virus was able to force density-arrested fibroblasts to re-enter the cell cycle, to traverse through S phase and enter mitosis. The presence of anaphase and telophase figures (Fig. 7B) even suggested that some cells, infected by the UL21a deletion virus, were able to divide. Thus, Cyclin A2 provides the missing link between virus-induced G1/S-promoting [32], [34], [38], [81] and G2/M-promoting activities [47], [82] that alone were unable to drive cell cycle progression to completion. Of particular interest in this context is the fact that Hertel and Mocarski have already described a “pseudomitotic” phenotype in the late phase of HCMV infection. This is characterized by an upregulation of mitosis-related gene expression and formation of abnormal mitotic spindles in the presence of an intact nuclear envelope [82]. This phenotype fits very well to previous findings by Sanchez *et al* showing HCMV-mediated activation but also cytoplasmic sequestration of the mitotic kinase Cyclin B1-CDK1 [47], whose nuclear translocation is an absolute prerequisite for nuclear envelope breakdown in prophase [63]. Considering that Cyclin A2 induction by UL21a-RXL2 mutation overcome these limitations (Fig. 6), Cyclin A2 clearly appears to be the rate-limiting factor for mitosis entry in HCMV-infected cells.

Primary, non-transformed cells possess potent surveillance mechanisms that arrest the cell cycle in response to deregulated DNA replication [83]. Similarly, entrance into mitosis is blocked if DNA replication is not completed [84]. Unscheduled cellular DNA synthesis (Fig. 4) and premature chromatin condensation (Fig. 5B) in UL21a-RXL-mutant-infected cells indicate that HCMV overrides these checkpoints, most likely by abrogating p53 and Chk2-dependent signaling [85], [86]. This and the partitioning of DNA repair enzymes to viral replication compartments predisposes the host cells to the accumulation of DNA damage [49], [87], [88]. It is yet unclear to what extent the intrinsic genotoxicity of HCMV contributes to the severe chromosomal instability seen after mitotic entry of infected cells. However, it is remarkable that checkpoint bypass by dual deficiency of p53 and Chk1 was recently shown to result in a mitotic catastrophe that closely resembles the extensive fragmentation of chromosomes and centromeres in HCMV-UL21a-RXL2mut-infected cells [89], [90]. An alternative explanation is that the prolonged mitotic arrest gives rise to the progressively increasing chromosomal damage seen in these cells [91]. This view is supported by the simultaneous loss of sister chromatid cohesion in many cells (Fig. 9), which is also considered as a characteristic result of extended metaphase arrest [92], [93]. Regardless to what extent the different mechanisms contribute to the severe chromosomal aberrations induced by the HCMV-UL21a-RXL mutant, the inherent capacity of this virus to promote both cell cycle progression and genetic instability may fuel the ongoing discussion about its potential role in cancer development and progression [94], [95].

## Materials and Methods

### Ethics statement

All procedures involving animals and their care were approved (protocol class 003-08/13-01/14; number 2170-24-01-13-03) by the Ethics Committee for Biomedical Research of the University of Rijeka Faculty of Medicine (Croatia) and were conducted in compliance with institutional guidelines as well as with national (Animal Protection Act 135/06 and 37/13; Regulations on the Protection of Animals Used for Scientific Purposes 55/2013) and international (Directive 2010/63/EU of the European Parliament and of the Council of 22 September 2010 on the protection of animals used for scientific purposes; *Guide for the Care and Use of Laboratory Animals*, National Research Council, 1996) laws and policies. Animals were bred and raised at the Laboratory for Mouse Breeding and Engineering Rijeka (LAMRI), University of Rijeka Faculty of Medicine. The authorization for breeding of and experiments on laboratory mice in this facility has been obtained by the competent national body (Veterinary Department of the Croatian Ministry of Agriculture – authorization No. HR-POK-004).

### Cells and viruses

Human embryonic lung (HEL) fibroblasts (Fi301, obtained from the Institute of Virology, Charité, Berlin, Germany) and human embryonic kidney (HEK) 293 cells (obtained from the Leibniz Institute DSMZ – *German Collection of Microorganisms and Cell Cultures*, Braunschweig, Germany) were maintained as described previously [52]. Where indicated, cells were treated with 2.5 µM MG132 or 50 ng/ml nocodazole (20 h before harvest) or were labeled with 10 µM 5′-bromo-2′-deoxyuridine (BrdU) for 1 h before harvest. Viruses were derived from the HCMV strain TB40-BAC4 [96] and modified by bacterial artificial chromosome (BAC) mutagenesis (see below). Viruses were propagated on HEL fibroblasts and infectious titers were determined as follows. Fibroblasts were seeded on 6-well plates and grown to confluence. Then, cells were infected with a series of virus dilutions in 0.5 ml of growth medium per well. The virus inocula were replaced by fresh culture medium after an adsorption period of 1 h. Cells were harvested at 24 hpi by trypsinization, counted with a hemocytometer and permeabilized with ice-cold PBS/80% ethanol for at least 5 min. After washing with PBS/1% BSA, cells were stained with an Alexa Fluor 488-conjugated IE1/2-specific antibody (clone 8B1.2, Merck-Millipore). The percentage of IE-positive cells was determined by flow cytometry. Only virus dilutions resulting in 1–10% IE-positive cells were used to calculate virus titers. This was done by multiplying the percentage of IE-positive cells by total cell number and dilution factor. For experiments, a multiplicity of infection (MOI) of 5–10 IE-protein forming units per cell was used. Fibroblasts were grown to confluence before infection to synchronize them in early G1 phase.

### BAC mutagenesis

A traceless mutagenesis method [97] was used to generate TB40-UL21a-RXL2mut, AD169-UL21a-RXL2mut, TB40-UL21a-APCmut and TB40-ΔUL21a. In brief, a kanamycin resistance cassette (KanR) was amplified from the plasmid pEPkanS2 [97], using the primer pairs UL21a-RRLFQmut-fw/rev, UL21a-PRmutAA-fw/rev, UL21a-del-fw/rev (table 1). The PCR products were transformed into Escherichia coli GS1783 [97], containing either TB40-BAC4 or AD169-pHB5 (both kindly provided by Jens von Einem, Ulm, Germany), and selected for integration of KanR. Subsequently, the KanR was removed by induction of λRed-recombinase and I-SceI expression. The HCMV-TB40-UL21a-RXL2 mutation was reverted using the primer pair UL21a-RRLFQ-revert-fw/rev (table 1). All steps of BAC mutagenesis were controlled by PCR and sequencing of the modified region. BACs were prepared and purified over NucleoBond Xtra-Midi columns (Macherey-Nagel) following the manufacturer's instructions. To reconstitute recombinant virus, the BACs were cotransfected with pp71/pUL82 and Cre recombinase expression plasmids into HEL fibroblasts by Amaxa nucleofection (Lonza).

**Table 1:** Oligonucleotide primers used in this study.

Primer name	Primer sequence (orientation 5′-3′, Pho: 5′ phosporylation)
UL21a-RRLFQmut-fw	GCCCTTGCGCCGCTTGGCTTTCTACGCGCCGCGAGCTCGTGCGCGCGCCTTCCAGAATCATATACATCCTAGGGATAACAGGGTAATCGATTT
UL21a-RRLFQmut-rev	CCAGCACTCGGCGCTGTTCTGGATGTATATGATTCTGGAAGGCGCGCGCACGAGCTCGCGGCGCGTAGAGCCAGTGTTACAACCAATTAACC
UL21a-RRLFQ-revert-fw	GCCCTTGCGCCGCTTGGCTTTCTACGCGCCGCGAGCTCGTCGGAGGCTTTTCCAGAATCATATACATCCTAGGGATAACAGGGTAATCGATTT
UL21a-RRLFQ-revert-rev	CCAGCACTCGGCGCTGTTCTGGATGTATATGATTCTGGAAAAGCCTCCGACGAGCTCGCGGCGCGTAGAGCCAGTGTTACAACCAATTAACC
UL21a-PRmutAA-fw	TGTTCCCCCCCATGTACCCGGTTTTGCTCCCTACCGCGTCGCCGCTCCCCACCCCATGATTCCCGATAGGGATAACAGGGTAATCGATTT
UL21a-PRmutAA-rev	AAAACTGGTCCCAATGTTCTTCGGGAATCATGGGGTGGGGAGCGGCGACGCGGTAGGGAGCAAAACGCCAGTGTTACAACCAATTAACC
UL21a-del-fw	AGTCATGCAGCGTGCGGCGCCTCTCTCATGGATCCACTGTCACCGTCGCGTAGGGATAACAGGGTAATCGATTT
UL21a-del-rev	ATATAGACTTTTATATGATCCCTGTACAGATGTAAATAAAATGTTTTTATCGCGACGGTGACAGTGGATCCATGAGAGAGGCGCCGCACGCTGCATGACTGCCAGTGTTACAACCAATTAACC
UL21a-fw-BamH1	CGTAGGATCCTATGGGAGGTAGCCCTGTTCC
UL21A-rev-Sal1	CGTAGTCGACAAACTGGTCCCAATGTTCTTC
UL21a-RXL1-ARAAF-BssH	Pho-GCTCGCCGGCCCTTGGCGCGCGCGGCTTTCTACGCGCC
UL21a-RXL1-rev	Pho-CCGAAAGTCCATGCGCACAGATGG
UL21a-RXL2-ARAFQ-BssH	Pho-GCGCCGCGAGCTCGTGCGCGCGCCTTCCAGAATCATATAC
UL21a-RXL2-rev	Pho-GTAGAAAGCCAAGCGGCGCAAGGG
UL21a-APC-AA-NarI	Pho-GCTCCCTACCGCGTCGCGGCGCCCCACCCCATGATTC
UL21a-APC-rev	Pho-AAAACCGGGTACATGAGGTGGAACATCGTCCAGC
CyclinA2-fw	AACAGCCAGACATCACTAACA G
CyclinA2-rev	TCAAACTTTGAGGCTAACAGCA
APC5-fw	AACGATTTCCGCCTAATAGCT
APC5-rev	AACCGCTTTCCTATAAACACC
GAPDH-fw	TTCACCACCATGGAGAAG
GAPDH-rev	CACACCCATCACAAACATGG

### Plasmids

The pUL21a coding sequence was amplified from TB40-BAC4, using primers UL21a-fw-BamH1 and UL21a-rev-Sal1 (see table 1). After BamH1/Sal1-mediated insertion into the prokaryotic expression vector pET-52b(+) (Merck), the resulting construct pET-52b(+)-UL21a served as template for site-directed inverse PCR mutagenesis. To introduce the RXL1 and RXL2 mutations, the 5′-phosphorylated primer pairs UL21a-RXL1-ARAAF-BssH/UL21a-RXL1-rev and UL21a-RXL2-ARAFQ-BssH/UL21a-RXL2-rev were used. The amplified, nicked plasmids were incubated with T4 DNA Ligase for 1 h at room temperature before being transformed into the Escherichia coli strain XL1-blue. UL21a-WT, UL21a-RXL1mut and UL21a-RXL2mut cDNAs were subcloned from pET52b(+) into the eukaryotic expression vector pCI-neo (Promega) in-frame to an N-terminal triple-hemagglutinin (3HA) epitope tag. To introduce a point mutation (PR^AA^) in the APC/C binding site (UL21a-APCmut), the pCI-neo-3HA-UL21a-WT plasmid was subjected to site-directed mutagenesis (see above) using the 5′-phosphorylated primers UL21a-APC-AA-NarI and UL21a-APC-rev (see table 1). All plasmids were confirmed by sequencing and purified by CsCl-ethidium bromide equilibrium centrifugation.

### His-pull down experiments

The pET-52b(+)-derived expression vectors were transformed into the Escherichia coli strain BL21-CodonPlus (DE3)-RILP (Stratagene). Recombinant Strep-pUL21a-His and Strep-pp150c-His proteins were expressed, purified and used for His-pull down assays as previously described [53], except for the following modifications: (i) pUL21a expression was induced by addition of isopropyl-β-D-1-thiogalactopyranoside (IPTG) to a final concentration of 0.5 mM; ii) after binding to Ni-NTA beads, pUL21a protein was washed 7 times with buffer containing increasing concentrations (up to 200 mM) of imidazole.

### Kinase assays

Kinase assays were performed as described, using recombinant pp150c as substrate [53]. Where indicated, equal amounts of either pUL21a-RXL2 or pUL21a-WT protein was added to the kinase reactions.

### Generation of monoclonal pUL21a antibody

Two BALB/c mice were immunized by subcutaneous injection of 50 µg of purified Strep-pUL21a-His protein in complete Freund's adjuvant. After three weeks, two mice were boosted with 50 µg of the same protein in complete Freund's adjuvant by injecting two-third volume subcutaneously and one-third volume intraperitoneally (i.p.). After two weeks, the sera of both animals were tested by ELISA for antibody titer against the recombinant pUL21a immunogen and the better responder was additionally boosted i.p. with 50 µg of the same protein dissolved in PBS. Three days later, spleen cells were collected and fused with SP2/O myeloma cells (ATCC: CRL 1581) at a ratio of 1∶1. The cells were seeded on 96-well tissue-culture plates in 20% Roswell Park Memorial Institute (RPMI) 1640 medium containing hypoxanthine, aminopterine, and thymidine for hybridoma selection. Supernatants of the generated mother-well cell lines were screened for antibodies, reactive against pUL21a immunogen by ELISA. Positive mother 4G12 was further cloned to generate UL21.02 cell line secreting monoclonal antibodies against pUL21a.

### Immunoprecipitations

The pCI-neo-3HA-UL21a expression plasmids were transfected into proliferating HEK293 cells using the Turbofect reagent (Fermentas). To stabilize pUL21a, MG132 was added to the cell culture medium at 24 h post transfection. Cells were harvested at 48 h post transfection and extracted by freezing-thawing in immunoprecipitation buffer (IPB): 50 mM Tris–Cl pH 7.4, 150 mM NaCl, 10 mM MgCl_2_, 10 mM NaF, 0.5 mM Na_3_VO_4_, 0.5% Nonidet P-40, 10% glycerol, 1 mM dithiothreitol (DTT), 2 g/ml aprotinin, 1 mM leupeptin, 1 mM Pefabloc. Cyclin A2-containing protein complexes were immunoprecipitated by incubating extracts for 2 h with agarose-conjugated Cyclin A2 antibodies (sc-751 AC, Santa Cruz). After 4 washing steps with IPB, the agarose-bound proteins were analyzed by immunoblotting for the presence of Cyclin A2, CDK2 and HA-tagged pUL21a.

### Subcellular fractionation

Nuclear and cytoplasmic fractions were prepared as described previously [53].

### Immunoblot analysis

Immunoblot analysis was carried out as described [52], using antibodies against IE1 (6E1, Vancouver Biotech), IE2 (clone 12E2, Vancouver Biotech), GAPDH (clone 6C5, Santa Cruz), HA (clones 12CA5 and 3F10, Roche), Cyclin A2 (clone BF683, BD Biosciences), Cyclin B1 (clone GNS1, Santa Cruz), Cyclin D1 (clone EPR2241, Abcam), Cyclin E1 (clone H-12, Santa Cruz), CDK1 (PC25, Merck-Calbiochem), CDK2 (clone 55, BD Biosciences), APC5 (A301-026A, Bethyl Laboratories), Lamin A/C (clone 636, Santa Cruz), β-Tubulin (clone 2-28-33, Sigma-Aldrich), pp65 (clone CH-12, Santa Cruz), pp28 (clone 5C3, Santa Cruz), pp150 (clone XP1, a gift of Bodo Plachter). The Strep-tag HRP detection kit (IBA Lifesciences) was used to detect bacterially expressed Strep-pUL21a-His proteins.

### Ribonuclease protection assay

Multi-probe ribonuclease protection assays were performed as described earlier [38]. The template set hCyc2 (BD Biosciences) was used for *in vitro* transcription of the radioactively labeled probe.

### Quantitative real-time PCR

Total cellular RNA was prepared, quantified, reverse-transcribed and analyzed by real-time PCR as described [51]. To determine mRNA expression levels of Cyclin A2, APC5 and GAPDH, the primer pairs cyclinA2-fw/cyclin A2-rev, APC5-fw/APC5-rev and GAPDH-fw/GAPDH-rev were used (see table 1).

### Flow cytometry

Cells were fixed, permeabilized and co-stained with propidium iodide and fluorescently labeled antibodies as previously described [52]. The following antibody combination was used: Alexa Fluor 488-conjugated anti-IE1/2 (clone 8B1.2, Merck-Millipore), anti-pH3(ser10) (clone 6G3, Cell Signaling) and BD Horizon V450-conjugated rat anti-mouse IgG_1_ (clone A85-1, BD Biosciences).

### Immunofluorescence microscopy

HEL fibroblasts were grown to confluency on glass coverslips before infection. After harvest, the cells were washed with PBS, fixed with 4% paraformaldehyde in PBS, permeabilized with PBST (PBS, 0.1% Triton X-100, 0.05% Tween 20), blocked and immunostained essentially as described elsewhere [98]. To enable immunofluorescence detection of chromosome-bound IE1 in mitosis (Fig. 6B, Fig. 10, Fig. S5), cells were fixed and permeabilized with ice-cold methanol according to Mücke et al [69]. To enable immunofluorescence microscopy of chromosome spreads, cells were swelled after harvest in hypotonic buffer (75 mM KCl) for 15 min at 37°C. Using a Cellspin I centrifuge (Tharmac), aliquots of 2.5×10^4^ cells were then cytocentrifuged at 250 g for 10 min onto a 6 mm×6 mm square sample area of Superfrost Plus glass slides (Thermo Scientific). After attachment, cells were allowed to dry for 15 min. Subsequently, cells were fixed with 4% paraformaldehyde for 10 min at room temperature and subjected to immunostaining. Primary antibodies against CENP-A (clone 3–19, GeneTex), pH3-ser10 (clone 6G3, Cell Signaling), Lamin A/C (clone 636, Santa Cruz), α-Tubulin (clone YL1/2, Merck Millipore), pUL44 (clone CH16, Santa Cruz), IE1/2 (clone 8B1.2, Merck Millipore), IE1 (clone 6E1, Vancouver Biotech) and IE2 (clone 12E2, Vancouver Biotech) were used. To allow various combinations of mouse and rat primary antibody clones, highly cross-absorbed anti-mouse IgG and IgG isotype-specific antibodies (Life Technologies) were employed as secondary reagents. For instance, the staining approach used in Fig. 6B included three different pairs of mouse primary and isotype-specific secondary antibodies (anti-IE1/2 + anti-mouse IgG2a-Alexa 488, anti-Lamin A/C + anti-mouse IgG2b-Alexa 594, anti-pH3-ser10 + anti-mouse IgG1-Alexa 647). Interphase nuclei and mitotic chromosomes were always counterstained by the use of 4′,6-diamidin-2-phenylindol (DAPI). Images were acquired by an Eclipse A1 laser-scanning microscope, using NIS-Elements software (Nikon Instruments). Equal microscope settings and exposure times were used to allow direct comparison between samples.

### Fluorescence in situ hybridization (FISH) and Giemsa staining

Giemsa staining was performed essentially as described [80]. Fluorescence *in situ* hybridization (FISH) was performed with whole chromosome painting (WCP) probes (Metasystems) for chromosome 1 (spectrum orange-labeled) and chromosome 3 (spectrum green-labeled) following the manufacturer's instructions with slight modifications.

## Supporting Information

Figure S1 **The Cyclin A2 interaction motif of pUL21a motif is required for viral inhibition of cellular DNA synthesis.** (A) Time schedule. Confluent fibroblasts were infected with HCMV. At 24 h post infection (hpi), cells were either treated with 0.5 mM foscarnet (PFA) or left untreated. Cells were harvested at 24, 48 and 72 hpi and analyzed for IE protein expression and DNA content by flow cytometry. (B) Cell cycle profiles of IE-positive cells are shown as DNA histograms. The positions of G0/G1 and G2/M phase cells are indicated by their 2n and 4n DNA content (n: haploid number of chromosomes).
(TIF)

Figure S2 **Flow cytometry gating strategy used to analyze cell cycle distribution and chromatin condensation of HCMV-infected cells.** Cells were stained with propidium iodide (PI), an Alexa Fluor 488-conjugated IE1/IE2 antibody and a combination of phospho-Histone H3(Ser10)-specific primary and BD Horizon V450-conjugated anti-mouse IgG_1_ secondary antibodies. The first two-parameter dot plot displays the forward and sideward light scatter (FSC and SSC) properties of flow cytometric events (upper left diagram). A region (P1) was set that excludes cellular debris and larger cell aggregates from further analysis. On a second dot blot (upper right diagram), cells from the P1 region were analyzed for area (A) and width (W) values of their PI fluorescence signal (recorded on the PerCP channel). The region P2 was set to gate out cell doublets. The third dot plot (middle left diagram) was used to analyze cells from the P2 region for IE1/IE2 protein expression (Alexa Fluor 488 signal, recorded on the FITC channel). An IE-positive cell population was defined by region P3. On the final dot plot (middle right diagram), cells from P3 were analyzed for DNA content (PI signal, recorded on the PerCP channel) and Histone H3-serine 10 phosphorylation (V450 signal, recorded on the Pacific Blue channel). The region P4 was set to calculate the percentage of IE-positive cells with condensed chromatin (H3-serine 10-positive fraction). Cells from the P4 region were highlighted in red, also within the parental regions. The gating hierarchy as well as the absolute and relative number of events in the four defined regions are displayed in the lower left table. The lower right panel shows the DNA histogram of IE-positive cells from region P3.
(TIF)

Figure S3 **Upregulation of Cyclins A2 and B1 as well as the induction of mitosis are specific consequences of the pUL21a-RXL2 point mutation.** The HCMV-TB40-UL21a-RXL2 revertant virus (RXL2rev) was compared to the parental wild type (wt) and UL21a-RXL2-mutant (RXL2mut) viruses with regard to Cyclin A2 and B1 protein expression and mitotic chromatin condensation. (A) Immunoblot analysis of whole cell lysates 48 h and 72 h after infection of density-arrested fibroblasts. (B) Flow cytometry of cellular DNA content and Histone H3-serine 10 phosphorylation at 72 h post infection. According to the gating strategy in Fig. S1 only the results of IE-positive cells are shown.
(TIF)

Figure S4 **The effects of UL21a-RXL2 mutation on cell cycle progression and virus growth are not HCMV strain specific.** Density arrested fibroblasts were infected with HCMV-AD169-UL21a-RXL2mut (MOI = 5). (A) Cells were harvested at regular intervals and analyzed by flow cytometry for DNA content, IE1/IE2 expression and histone H3(ser10) phosphorylation according to Fig. S2. Shown are DNA/pH3(ser10) dot plots of IE1/IE2 positive cells at 3 dpi when the number of mitotic, pH3(ser10)-positive events reached a maximum; n: haploid number of chromosomes. (B) At the indicated time points, cell culture supernatants were analyzed in biological triplicates for the number of IE protein-forming units (IU) by virus titration. Mean values and standard deviations are indicated in the virus growth curves.
(TIF)

Figure S5 **Analysis of nucleo-cytoplasmic distribution of pUL21a and Cyclin A2.** Density arrested human embryonic lung fibroblasts were infected with HCMV reconstituted from TB40-BAC4-wt or derivatives carrying the indicated UL21a mutations. To facilitate detection of pUL21a and Cyclin A2, the proteasome inhibitor MG132 was added at 48 hpi to a final concentration of 2.5 µM. Nuclear and cytoplasmic fractions were prepared and analyzed by immunoblotting for the presence of Cyclin A2, pUL21a, Lamin A/C (nuclear marker) and β-Tubulin (cytoplasmic marker). The wt and mutant forms of pUL21a were found to be expressed at similar levels and to be present in both nuclear and cytoplasmic fractions. Nuclear localization of Cyclin A2 was dependent on the absence of an intact pUL21a -RXL2 motif.
(TIF)

Figure S6 **Loss of centromeres occurs in HCMV-UL21a-RXL2mut-infected cells.** Fibroblasts were seeded on coverslips and grown to confluence. Subsequently, cells were infected with HCMV-wt or HCMV-UL21a-RXL2mut as indicated. At 72 hpi cells were fixed with methanol and examined by immunofluorescence microscopy for DNA (DAPI staining), IE gene expression (IE1/2 staining) and localization of centromeres (CENP-A staining). Representative images are shown. All visible cells are IE-positive. Condensed chromosomal material lacking centromeres and accordingly has accumulated at the periphery of mitotic cells is marked by arrowheads.
(TIF)
