# Supplementary figures and images for: PUL21a-Cyclin A2 Interaction is Required to Protect Human Cytomegalovirus-Infected Cells from the Deleterious Consequences of Mitotic Entry
Source: PLoS Pathog. 2014 Nov 13;10(11):e1004514. doi: 10.1371/journal.ppat.1004514 (PMC4231158; doi:10.1371/journal.ppat.1004514)

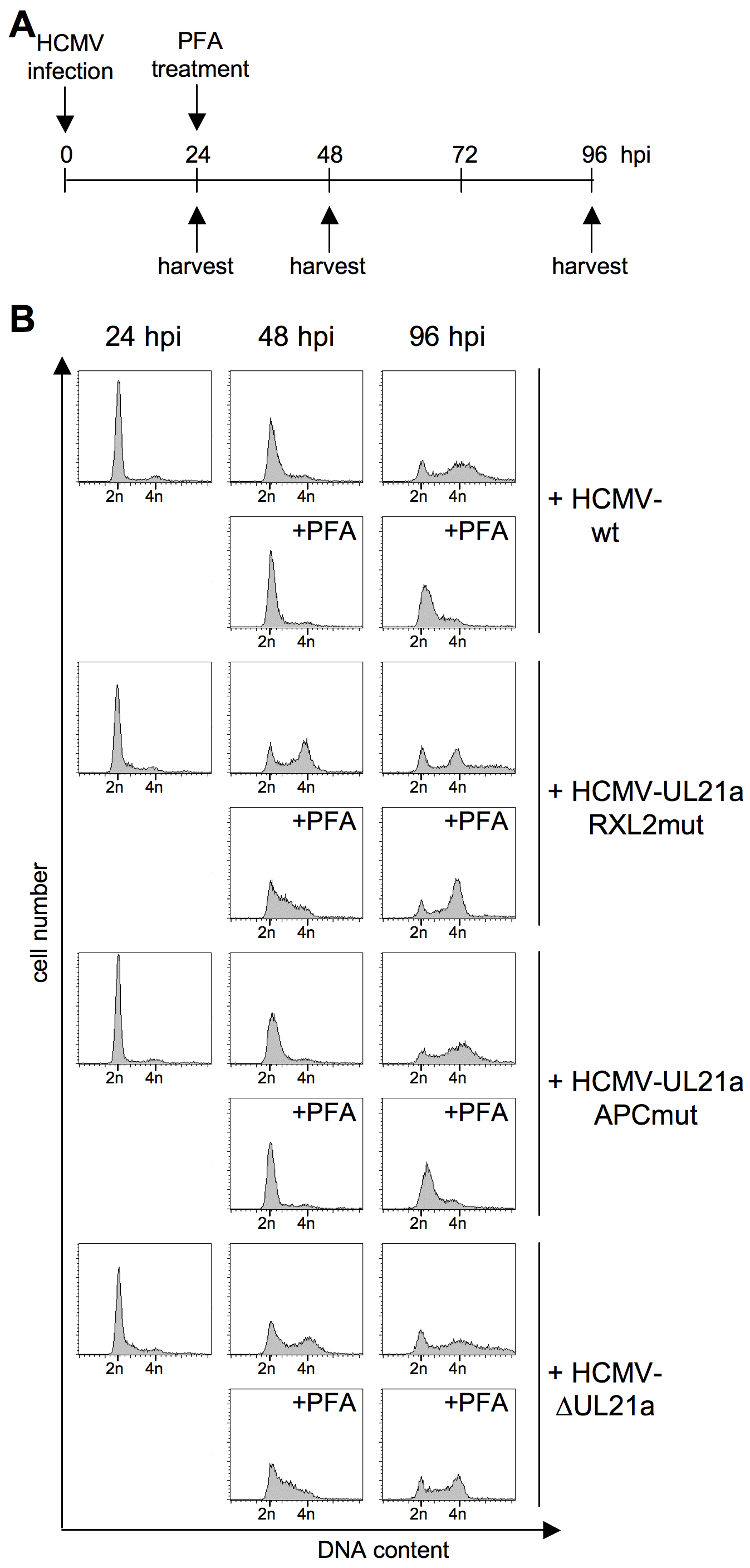

Supplement: Figure S1 — The Cyclin A2 interaction motif of pUL21a motif is required for viral inhibition of cellular DNA synthesis. (A) Time schedule. Confluent fibroblasts were infected with HCMV. At 24 h post infection (hpi), cells were either treated with 0.5 mM foscarnet (PFA) or left untreated. Cells were harvested at 24, 48 and 72 hpi and analyzed for IE protein expression and DNA content by flow cytometry. (B) Cell cycle profiles of IE-positive cells are shown as DNA histograms. The positions of G0/G1 and G2/M phase cells are indicated by their 2n and 4n DNA content (n: haploid number of chromosomes). (TIF) [file ppat.1004514.s001.tif]

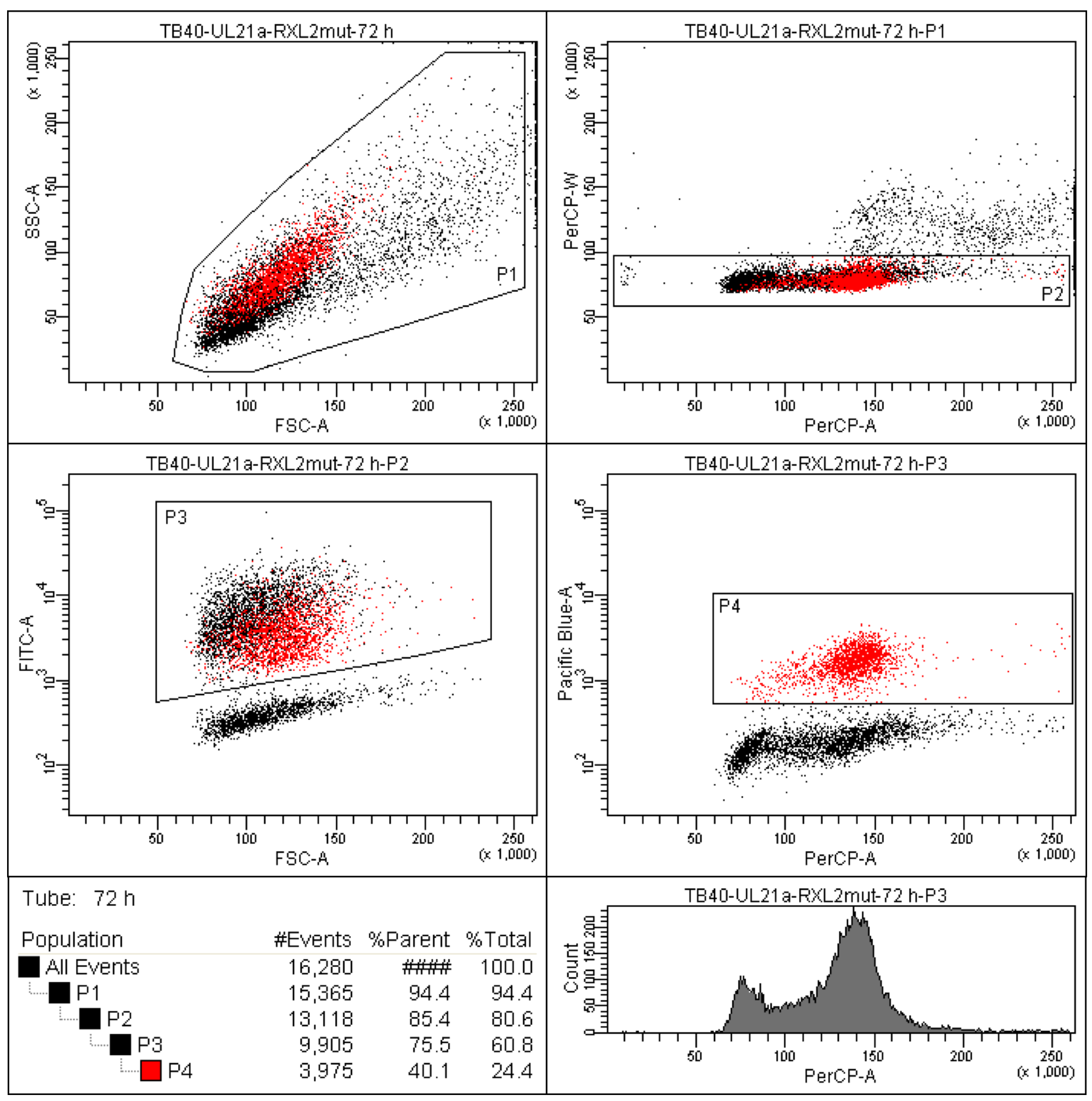

Supplement: Figure S2 — Flow cytometry gating strategy used to analyze cell cycle distribution and chromatin condensation of HCMV-infected cells. Cells were stained with propidium iodide (PI), an Alexa Fluor 488-conjugated IE1/IE2 antibody and a combination of phospho-Histone H3(Ser10)-specific primary and BD Horizon V450-conjugated anti-mouse IgG1 secondary antibodies. The first two-parameter dot plot displays the forward and sideward light scatter (FSC and SSC) properties of flow cytometric events (upper left diagram). A region (P1) was set that excludes cellular debris and larger cell aggregates from further analysis. On a second dot blot (upper right diagram), cells from the P1 region were analyzed for area (A) and width (W) values of their PI fluorescence signal (recorded on the PerCP channel). The region P2 was set to gate out cell doublets. The third dot plot (middle left diagram) was used to analyze cells from the P2 region for IE1/IE2 protein expression (Alexa Fluor 488 signal, recorded on the FITC channel). An IE-positive cell population was defined by region P3. On the final dot plot (middle right diagram), cells from P3 were analyzed for DNA content (PI signal, recorded on the PerCP channel) and Histone H3-serine 10 phosphorylation (V450 signal, recorded on the Pacific Blue channel). The region P4 was set to calculate the percentage of IE-positive cells with condensed chromatin (H3-serine 10-positive fraction). Cells from the P4 region were highlighted in red, also within the parental regions. The gating hierarchy as well as the absolute and relative number of events in the four defined regions are displayed in the lower left table. The lower right panel shows the DNA histogram of IE-positive cells from region P3. (TIF) [file ppat.1004514.s002.tif]

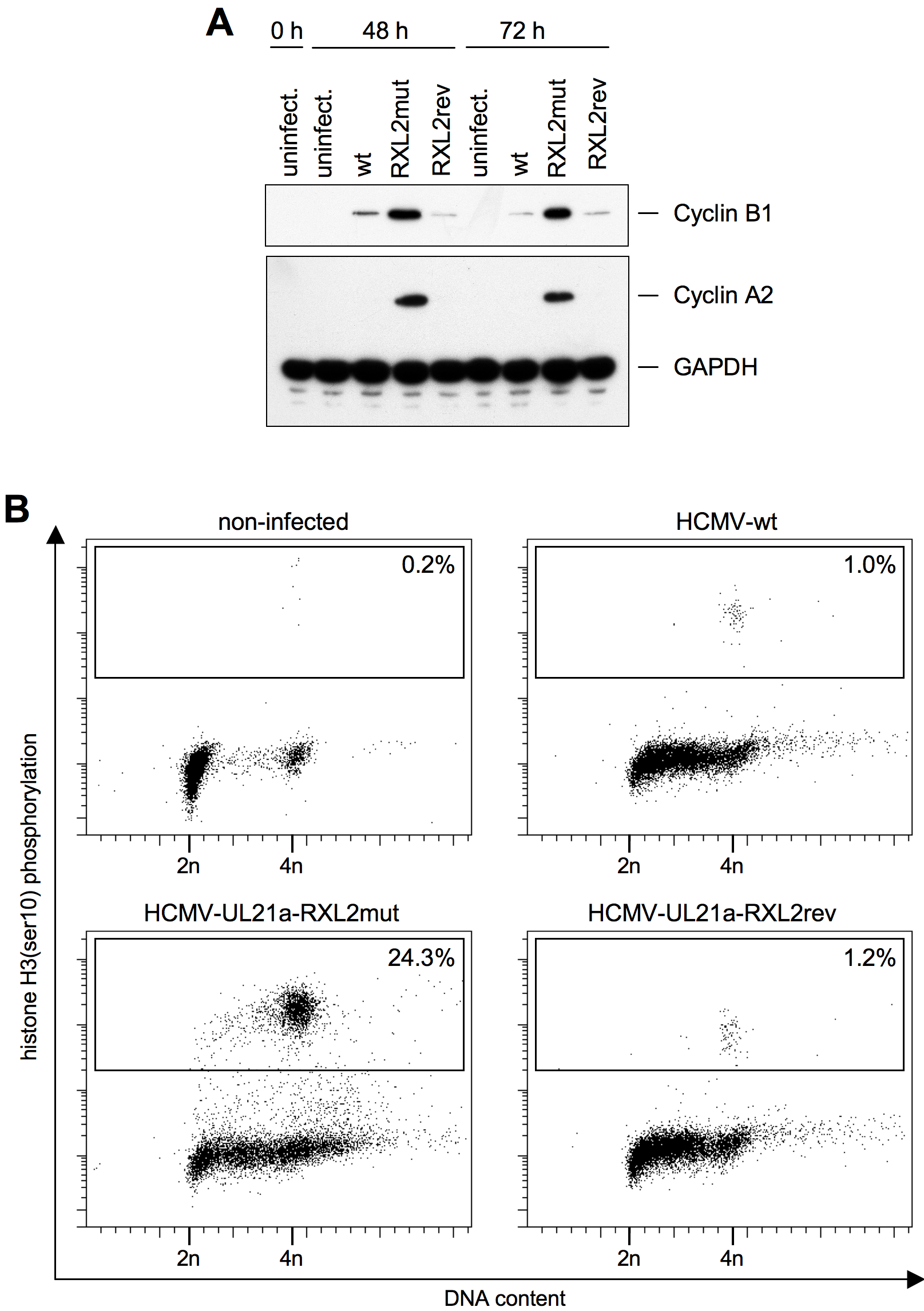

Supplement: Figure S3 — Upregulation of Cyclins A2 and B1 as well as the induction of mitosis are specific consequences of the pUL21a-RXL2 point mutation. The HCMV-TB40-UL21a-RXL2 revertant virus (RXL2rev) was compared to the parental wild type (wt) and UL21a-RXL2-mutant (RXL2mut) viruses with regard to Cyclin A2 and B1 protein expression and mitotic chromatin condensation. (A) Immunoblot analysis of whole cell lysates 48 h and 72 h after infection of density-arrested fibroblasts. (B) Flow cytometry of cellular DNA content and Histone H3-serine 10 phosphorylation at 72 h post infection. According to the gating strategy in Fig. S1 only the results of IE-positive cells are shown. (TIF) [file ppat.1004514.s003.tif]

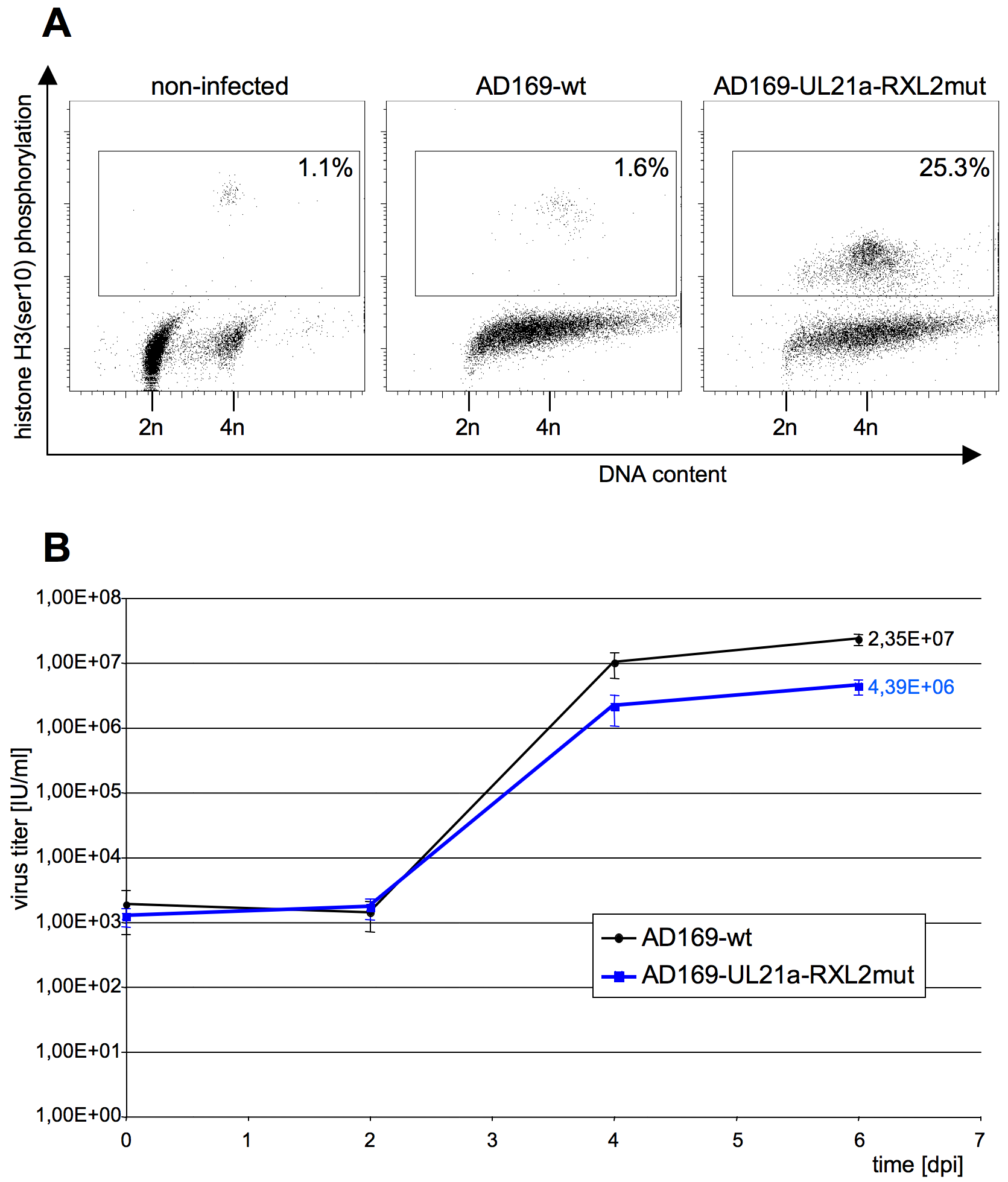

Supplement: Figure S4 — The effects of UL21a-RXL2 mutation on cell cycle progression and virus growth are not HCMV strain specific. Density arrested fibroblasts were infected with HCMV-AD169-UL21a-RXL2mut (MOI = 5). (A) Cells were harvested at regular intervals and analyzed by flow cytometry for DNA content, IE1/IE2 expression and histone H3(ser10) phosphorylation according to Fig. S2. Shown are DNA/pH3(ser10) dot plots of IE1/IE2 positive cells at 3 dpi when the number of mitotic, pH3(ser10)-positive events reached a maximum; n: haploid number of chromosomes. (B) At the indicated time points, cell culture supernatants were analyzed in biological triplicates for the number of IE protein-forming units (IU) by virus titration. Mean values and standard deviations are indicated in the virus growth curves. (TIF) [file ppat.1004514.s004.tif]

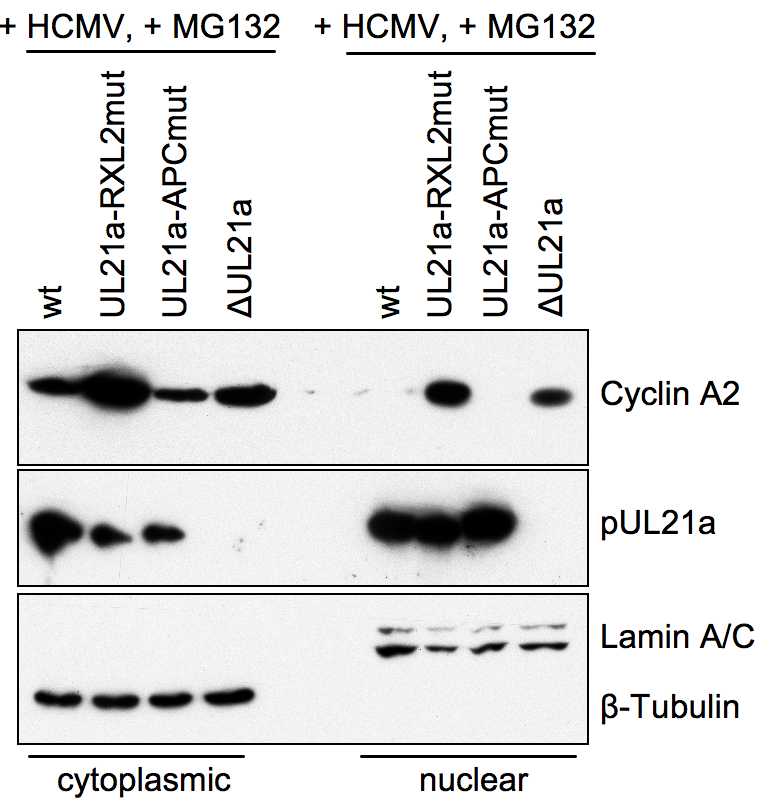

Supplement: Figure S5 — Analysis of nucleo-cytoplasmic distribution of pUL21a and Cyclin A2. Density arrested human embryonic lung fibroblasts were infected with HCMV reconstituted from TB40-BAC4-wt or derivatives carrying the indicated UL21a mutations. To facilitate detection of pUL21a and Cyclin A2, the proteasome inhibitor MG132 was added at 48 hpi to a final concentration of 2.5 µM. Nuclear and cytoplasmic fractions were prepared and analyzed by immunoblotting for the presence of Cyclin A2, pUL21a, Lamin A/C (nuclear marker) and β-Tubulin (cytoplasmic marker). The wt and mutant forms of pUL21a were found to be expressed at similar levels and to be present in both nuclear and cytoplasmic fractions. Nuclear localization of Cyclin A2 was dependent on the absence of an intact pUL21a -RXL2 motif. (TIF) [file ppat.1004514.s005.tif]

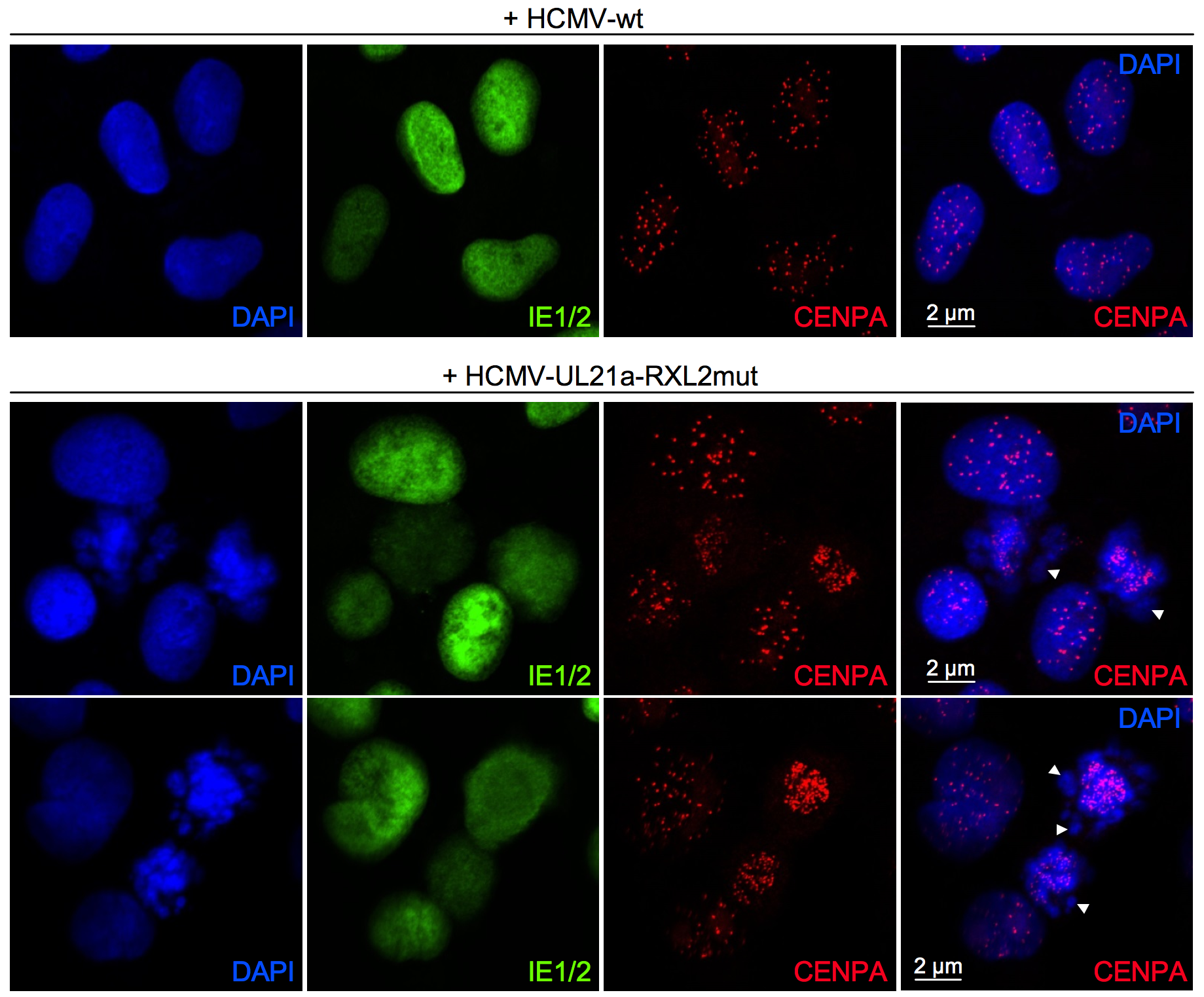

Supplement: Figure S6 — Loss of centromeres occurs in HCMV-UL21a-RXL2mut-infected cells. Fibroblasts were seeded on coverslips and grown to confluence. Subsequently, cells were infected with HCMV-wt or HCMV-UL21a-RXL2mut as indicated. At 72 hpi cells were fixed with methanol and examined by immunofluorescence microscopy for DNA (DAPI staining), IE gene expression (IE1/2 staining) and localization of centromeres (CENP-A staining). Representative images are shown. All visible cells are IE-positive. Condensed chromosomal material lacking centromeres and accordingly has accumulated at the periphery of mitotic cells is marked by arrowheads. (TIF) [file ppat.1004514.s006.tif]
